# *In vitro* analysis of quercetin-like compounds from mistletoe *Dendrophtho*e *pentandra* *(L.) Miq* as a potential antiviral agent for Newcastle disease

**DOI:** 10.12688/f1000research.133489.8

**Published:** 2024-07-23

**Authors:** Lazuardi Mochamad, Selvaraja Malarvili, Khairat Jasmine, Vuanghao Lim

**Affiliations:** 1 Sub-division Veterinary Pharmacy Science, Faculty of Veterinary Medicine, Universitas Airlangga, Surabaya, East Java, 60115, Indonesia; 2 Faculty of Pharmaceutical Sciences, UCSI University, No.1, Jalan Menara Gading, Taman Connaught, Kuala Lumpur, Wilayah Persekutuan, Malaysia; 3 Institute of Biological Science, Faculty of Science, University Malaya, Kuala Lumpur, 50603, Malaysia; 4 Advanced Medical and Dental Institute, Universiti Sains Malaysia, Bertam 13200 Kepala Batas, Penang, Penang, 13200, Malaysia

**Keywords:** Antiviral agents, Benalu Duku, Health life, Herbal medicine, Inactivant

## Abstract

**Background:**

Recent evidence suggests that some flavonoid compounds obtained from crude methanol extract of mistletoe leaves ( *Dendrophthoe pentandra L. Miq*), also known as Benalu Duku (BD), have antimicrobial effects. Thus, the plant has the potential to eliminate viruses that may cause outbreaks in chicken farms. This study aimed to prove the *in vitro* ability of flavonoid compounds, namely quercetin-like compounds (QLCs), to eliminate field viruses, specifically the Newcastle disease virus (NDV).

**Methods:**

This research was performed in two stages. An *in vitro* test was used with a post-test of the control groups designed at a significance of 0.05. BD leaves (5 kg) were extracted using a maceration method with methanol and then separated into hexane, chloroform, ethyl acetate, and methanol fractions. The final extracted products were separated using semi-preparative high-performance liquid chromatography (HPLC) to obtain QLCs. The QLCs were identified and compared with quercetin using HPLC, proton and carbon nuclear magnetic resonance spectrometry, Fourier transform infrared spectrophotometry and ultra-performance liquid chromatography-mass spectrometry. The activity of QLCs was tested *in vitro* against the NDV at a virulence titter of 10 ^−5^ Tissue Culture Infectious Dose 50% (TCID50) in chicken kidney cell culture.

**Results:**

Solutions of 0.05% (w/v) QLCs were discovered to have antiviral activity against NDVs, with an average cytopathogenic effect antigenicity at a 10 ^−5^ dilution (p<0.05).

**Conclusions:**

QLCs from flavonoids from the leaves of BD have *in vitro* antiviral bioactivity against NDV at a virulence titter of 10-5 Tissue Culture Infectious Dose 50% (TCID50) in chicken kidney cell culture. QLCs may have the potential to be developed as medicinal compounds for the treatment of other human or animal viral infections.

## Introduction

Mistletoe plants contain flavonoids; these compounds are often found in the leaves. The mistletoe *Dendrophthoe pentandra (L.) Miq.*, known in Indonesia as *Benalu Duku* (BD), has multiple benefits, especially for medicines intended to treat animal and human diseases. ^1^^,^^2^ Interestingly, reports stated that the leaf part of BD comprises mainly flavonoid elements with a polyphenol structure or analogous to quercetin, and are therefore expected to have antimicrobial potential. ^3^ Consequently, they may be antibacterial or antiviral compounds; thus, they show great promise for further development into candidates for new plant-based, or specifically mistletoe-based, antimicrobial compounds. ^4^ In terms of its antiviral properties, there are generally three main places where functional groups may cause these effects, namely complex aromatic structures, electron attractor groups, and quercetin analogs. ^5^^,^^6^ That concept can be explained using theory based on quantification structure-activity relationship versus virus agents. As an anti-virus, it is suitable for killing viruses that live and develop in host eukaryote cells that have the property of being able to regrow themselves against eukaryote cells that have been damaged such as intestinal cells, skin epithelial cells, epithelial cells of the reproductive tract. ^7^ This phenomenon can be more easily determined after initial *in vitro* screening in a group of viruses under aerobic conditions or living in hydrophilic cells, such as a laryngotracheal cell. ^8^ These compounds are said to be quercetin analogs because the bioactive separation of quercetin in plants is difficult and frequently contaminated with other compounds. ^9^ Thus, the main chemical and physical characteristics of the quercetin structure are retained in quercetin-like compounds (QLCs).

Some viruses can survive in laryngotracheal cells; one such virus is the Newcastle disease virus (NDV) that often affects poultry, especially chicken and birds. Infected cases primarily present as acute respiratory symptoms, then depression, and finally as nervous manifestations in late infection. ^10^ Diarrhea may be the predominant clinical symptom. ^11^ The severity of the infection is determined by the virulence of the infecting virus and the host susceptibility. ^11^^,^^12^ The occurrence of cases is reportable; they may result in trade restrictions. NDV is also known as avian paramyxovirus serotype 1 (PMV-1). NDV is an RNA virus with at least 11 pathogenic PMV serotypes in poultry. ^13^ The classification of NDV isolates is divided into three groups according to virulence: the malignant group (velogenic), the moderately malignant group (mesogenic), and the group with low malignancy (lentogenic). ^14^

The exploration of QLCs as an antimicrobial agent, especially an antiviral, is critical given the demands of the World Health Organization (WHO) and Food Agriculture Organization (FAO) regarding the third sustainable development goal (to ensure healthy lives and promote well-being for all at all ages) will be related to veterinary drug residues and antimicrobial resistance (AMR). ^14^^,^^15^ The selection of natural compounds from plants, such as QLCs, will minimize the risk of the adverse impacts of AMR and animal drug residues in livestock that will be consumed by both adults and children. ^16^^,^^17^ It is known that efforts to replace bioactive antivirals with synthetic compounds will impact the problem of veterinary drug residues; thus, efforts to use QLCs as a *Remedies Cardinal* formula for an antiviral drug are very useful. ^18^^,^^19^ The purpose of the study was to prove the ability of QLCs extracted from BD leaves to eliminate the NDVs that cause outbreaks in poultry. In this study, *in vitro* investigations were performed to determine the antiviral potential of bioactive compounds in BD leaves. The preliminary data produced will be continued with *in vivo* studies or field tests using bioactive formulations containing QLCs in the future.

## Methods

### Sample collections

BD plants were obtained from the south of Sumatera, in the district of Muara Enim, Indonesia, at the geographical coordinates of 3° 39′ 00″ S″, 103° 48′ 00″ E″. The plant, as mistletoe, is *Dendrophthoe pentandra (L.) Miq.*, was identified by the Directorate Control of the Natural Science Collection, National Bureau of Innovative Research, official letter number B-1679/11.6.2/DI.05.07/6/2022, at the address of Raya Jakarta – Bogor, Cibinong 16911, on June 7 ^th^, 2022, with the Indonesian name BD. Sampling began in March 2022 and lasted until August 2022.

### Viruses

The virus was identified as a NDV obtained from an infected chicken at a chicken breeding farm in Gresik District (East Java), Indonesia, in October 2022 (at the geographical coordinates of 7° 9′ 14″ south and 112° 39′ 22″ east). The seed virus was characterized as velogenic by referring to the veterinarian officer (VO) of that breeder and agreed upon by the VO and the head of the research team. The VO performed identification using a standard method field test model based on live virus vaccination. Identification and characterization of the ND field virus, which is the same type of virus that hit the duck outbreak in the same place one year ago. ^20^ A total of 30 male chickens aged 6 months were divided into six groups, and each group consisted of five chickens. Group 1 was given the name without vaccination (NV), group 2 was given the name vaccination Avian Encephalitis (AE), group 3 was named the vaccination infectious avian influenza (AI) vaccine, group 4 was given the name vaccination Infectious Laryngotracheitis (ILT), group 5 was given the name vaccination infectious bronchitis (IB), and group 6 was given the name vaccination Newcastle disease virus (NDV). The vaccines of AE, AI, ILT, IB, and NDV were used commercial inactivated vaccines. Of the six groups at 3 ^rd^-day post-vaccination, it turned out that the NV, AE, AI, ILT, and IB groups were infected with the NDV virus, with symptoms such as the head being often positioned downward towards the feet, drooling frequently, no appetite, pale eye mucosa, and body temperatures reaching 41°C. During the auscultation, there was noise and the sound of secretions. The group that had received the NDV vaccine was not infected with the virus. The Observation of the infected chicken was analyzed during the five days. From the five NV groups, saliva was collected and made free from bacterial and fungi or/and also parasites after being added to antibiotics (Penicillin G w/v Oxoid Pharma 1.500.000 UI catalog number: CT0043B; Kanamycin Injection Meiji Pharma v/v 50 mg/mL), anti-fungi (Nystatin Taisho Pharma w/v 100.000 IU), and anti-parasites (Metronidazole Medicina Interna Pharma w/v 50 mg/mL). Substances of NDV were performed as velogenic substances and characterized as follows; (a) pyrogen-free, (b) iso-tonic, (c) iso-ionic, and (d) iso-hydric. ^21^ Re-isolation of NDV after infection was carried out by initially using embryonated chicken eggs (9-11 days old) with Specific Pathogen Free (SPF) criteria obtained from a chicken breeding company in the city of Bogor-Indonesia. Three SPF eggs were washed clean and the presence of chicken embryos was observed. Next, the egg sac is punctured and 1 mL of NDV virus solution is injected. The injection site is closed using solidum paraffin. Next, the infected eggs are incubated for 48 hours, noting that every 24 hours, the chicken embryo must remain alive. The next step is to harvest the results of planting the virus suspension by taking the chorioallantois membrane of chicken embryos and washing them by adding a solution of sodium chloride sterile 0.9% (w/v) pH 6.80 at proportion fraction 1:5 and shaking slowly followed by cold centrifugation at 1500 rates per minute. The final stage of washing is that the sediment is added to Medium Eagle Media containing 10% Foetal Bovine Serum. ^22^ The supernatant was used for testing as a field virus, NDV, to be developed and propagated using Vero Cell culture in Dulbecco’s modified eagle’s medium (DMEM, Merck catalog number: D5030) added to 10% of Foetal Bovine Serum (FBS, Merck catalog number: 12003C). The substance of NDV was propagated into the Roux’s plastic bottle (250 mL contains 14×10 ^5^ Vero cells infected/mL), then harvested to obtain the virulence level of 10 ^−5^ Tissue Culture Infectious Dose 50% (TCID50).

### Sample preparations for Benalu Duku

Approximately 5 kg of BD as samples were washed using aqua demineralization (PT Brataco Surabaya-Indonesia catalog number: 01-Aquadem) and separated from other leaves to be pulverized using a pulverized apparatus (HBS Blender catalog number: MTA-94200747) so that the powder obtained weighed approximately 284 g. Pulverized BD was extracted by methanol pro analysis (p.a.) grade (Merck catalog number: 111457-85-3) using a movement maceration method for 48 h. The obtained raffinates were further concentrated using a rotary evaporation device (Buchi Rotavapor R-200 Rotary Evaporator) connected to a water bath apparatus adjusted at 40°C. Crude extract from Raffinate from methanol p.a. was added to sterile aqua demineralization (100 mL) and put in a partition separator flask (Iwaki Separator Flask 250 mL catalog number: 6401FS100). Further, ethyl acetate p.a. grade (Merck catalog number: 141-78-6) was added up to the top volume level. The results of tannin-free raffinate were further dried using nitrogen vapor in a water bath at 40°C (Funke Gerber Germany catalog number: WB 436). Thus, tannin-free raffinates were obtained. Tannin-free raffinates were further partitioned by workflow, added to the methanol p.a. grade, and repartitioned in a partition flask with n-hexane p.a. grade as a menstruum solution. The partitioned raffinate was dried using nitrogen vapor at pharmaceutical grade from PT Matesu Gotty-Surabaya (Indonesia), immersed in a water bath, and set at a temperature of 40°C. ^21^^,^^23^

### Qualitative flavonoid analysis

The raffinates were analyzed for flavonoids using two qualitative flavonoid test methods. In the first method, 1 mg of the obtained raffinates was randomly taken and 10 mL of methanol p.a. grade (Merck catalog number: 111457-85-3) was added until dissolved. Especially for raffinate solution, 0.6 mL of iron chloride (III) solution, 1% (w/v) (Merck catalog number: 7705-08-0), was added, causing a bluish color change as well as an indicator of flavonoid content in the extract. In the second method, 1 mg of dried raffinates was randomly taken and dissolved in 10 mL (w/v) methanol p.a.; then, 3 mL of the mixture was taken and placed in three 10 mL test tubes (control tube A, tube B color indicator, tube C test sample). Then, concentrated hydrochloride acid (HCl) (Merck catalog number: 109057) was prepared and 10 drops were placed in tube B and tube C. Tube B was heated by a burner for 5 min and became more concentrated. To tube C (test tube), zinc p.a. (Merck catalog number: 7440-66-6) 0.1 mg was added. The tube was left to stand for 5 min and the color change was observed in tube C compared with tube B and tube A; tube C appeared green, tube A was brown, and tube B was dark. The green discoloration of tube C indicated that flavonoids were present.

In the second method, 1 mg of the viscous extract was taken randomly and dissolved in methanol p.a., 10 mL (w/v). Quercetin dihydrate p.a. (Merck catalog number: 551600) was also prepared as a standard 1 mg dissolved in methanol p.a., 10 mL (w/v). The thin-layer chromatography (TLC) containing activated magnesium silicate was produced by Supelco Corp. (Catalog number: 1343-88-0). The lower limit was measured at 2 cm and marked as a line. Additionally, prepared in the chamber of the mobile phase solution was 1-butanol p.a. (Merck catalog number: 101990), acetic acid p.a. (Merck catalog number: 64-9-7) and sterile water ( *Perusahaan Terbatas Kimia Farma* Indonesia catalog number: 01-aquadest) in a 1:1:1 ratio. Dropwise, samples of raffinates were added to methanol p.a. and quercetin standard on the TLC paper, and elution was performed using the prepared mobile phase. The paper was taken and observed under UV light at a wavelength of 366 nm (yellow light). The calculation of the retention factor (Rf) between the two compounds was performed to aim to produce the same R _f_ value. The presence of flavonoids will be more noticeable when spraying ammonium solution p.a. (Merck catalog number 119812) on post-elution TLC, both sample drip and standard, with red color appearance indicators. The red color indicates the presence of flavonoids in raffinate. ^21^

### Optimizing the mobile phase for column chromatography to separate the quercetin-like compounds

In this stage of the work, the mobile phase was optimized by searching for the mixed fraction of the composition of the polar solution (solution contains bonds between atoms that have very different electronegativities, which cause a dipole moment) so that it could separate the analytes using the stationary phase for TLC. The mobile phase fraction sought was a mixture of water and methanol, water pro chromatograph (p.c.)–methanol p.c. so that it could later be applied to semi-preparative HPLC devices. In the search, the fractions water:methanol as follows; 4:6, 3:7, 2:8, and 1:9 were tested. The selected fraction of the mobile phase was used to separate the compounds of BD extract from impure compounds to obtain the QLCs. The implementation of the research began with the preparation of TLC and the preparation of the mobile phase solution. A mobile phase mixture of water p.c.-methanol p.c. was prepared in a chamber. TLC was outlined according to the area to be dripped, which was 2 cm from the bottom of the chamber. Furthermore, the chamber was added to the mobile phase solution with a note that the surface of the mobile phase solution does not exceed the line limit of 2 cm. Furthermore, chamber saturation was carried out by inserting filter paper and the chamber was closed followed by the dripping of raffinate (QLCs) and quercetin standards on the marked TLC. The next step was to eluate the TLC that has been dripped into the two substances for 30 minutes. The end of the elution was carried out by drying of TLC and spraying of ammonium solution. The results of the TLC were carried out Rf examination using UV TLC viewer equipment from Vilber (Spain), Lourmat TLC Viewing Chamber CN-15.LC 4121 4155 1 (at wavelengths 366 nm).

### Separation of quercetin-like compounds of Benalu Duku

Dried samples, as a crude extract compound at 39 g, were added to the methanol pro chromatograph, ready for purification of the obtained flavonoid compounds or polyphenol substances (carbon-3 and carbon-6). Chemical compounds for the separation of flavonoid compounds with semi-preparative HPLC are used with high purity or pro-chromatography at a purity of 99.99% of the active substance as methyl alcohol (CH _3_OH) or 99.9% of the active substance, namely pro-chromatographic water (H _2_O). The standard for identification of the polyphenol substances was quercetin p.a. grade at 99.97% purity of active substances as 2-(3,4-Dihydroxyphenyl)-3,5,7-trihydroxy-4H-1-benzopyran-4-one (C _15_H _10_O _7_).

The HPLC equipment used was a Shimadzu CBM-20A Communication Bus Module with a photodiode array detector in an ultraviolet–visible (UV–visible) M20A spectrometer, in which a preparative column (Xtendedlife diameter, 30 mm; length, 100 mm; particle size, 7 μm; catalog number 35007-109370XL) was fitted. The HPLC system was set to isocratic elution parameters: detection, 230–700 nm; flow rate, 0.5 mL; stop time, 20 min; mobile phase solution, 70% methanol pro chromatograph (p.c.) (Merck catalog number: 67-56-1): 30% water p.c. (Merck catalog number: 7732-18-5); loop injection capacity, 250 μL. In the second part of the experiment, the standard quercetin p.a. was dissolved (w/v) in the mobile phase solution at serial concentrations of 0.5, 0.75, and 1 μg/mL, then injected into the HPLC system in 0.5 μL aliquots. The crude raffinates from the workflow of the chromatography column were dissolved in the mobile phase solution, filtered using a 0.20 μm filter (Merck catalog number: CLS431212), and injected into the HPLC system. The sample chromatogram was compared with the standard chromatogram, quercetin p.a., based on retention time (RT), and the concentration of flavonoids in the sample was calculated based on the area of the standard chromatogram compared to the area of the sample chromatogram. In the third part, flavonoid compounds in matrix samples were isolated using analyte columns based on standard RT, and analytes from waste products were collected from HPLC equipment. ^23^^,^^24^

### Observation of analytes similar to standard compounds

The physicochemical properties of the two compounds were used to determine the analyte’s molecular structure similarity test (QLCS) in comparison to the reference molecular structure (quercetin), which was carried out using a physical-chemical-based test apparatus. The analysis of the phenomenon of adsorption–partition against the stationary phase octadecyl xylene (reverse-phase) was determined using HPLC. The HPLC specification used a similar workflow for the separation of QLCs from BD flavonoids. The column installed was an RP C _18_ 15-cm stainless steel with a diameter of 0.20 μm (Agilent product catalog number 5982-1111). As for the mobile phase, water p.c.–methanol p.c. was used with a composition of 30:70. The flow rate was set to 0.5 mL/min and the column temperature was set to 20°C. The detector was a photodiode array with settings of 190–900 nm. Observations were made by comparing the similarity of RT between QLCs and quercetin standards. ^23^^,^^25^^,^^26^

The analysis of QLCs compared with standards was by Fourier transform infrared (FT-IR) spectrophotometer transmission method by potassium bromide p.a. (Merck catalog number: 7758-02-3) observed using peak similarity ranging from 4000 cm ^−1^ to 400 cm ^−1^, percentage transmission (%T), 20 scans, at a resolution of 4 cm ^−1^. The instrument FT-IR Spectrum One Perkin Elmer (PN: 09934358) produced by LabMakelaar Benelux B.V. Knibbelweg 18C, NL-2761, JE Zevenhuizen (ZH) (The Netherlands) was used. The research workflow for FT-IR observations was prepared in the Faculty of Pharmacy Universitas Airlangga. The implementation of FT-IR begins with the manufacture of tablets by mixing analyte QLCs with Kalium bromide (KBr) powder and mashing them in a porcelain mortar. Furthermore, it was printed into a tablet using a tablet printer device, and the formed tablet was placed on the basket of the FT-IR device and immediately checked by first setting the wave number according to the specifications of the FT-IR device. The technique was also carried out on a standard compound (quercetin). ^6^^,^^27^^,^^28^

Proton nuclear magnetic resonance ( ^1^H NMR) and carbon nuclear magnetic resonance ( ^13^C NMR) were performed using a JEOL NMR (JNM ECS-400, 400 MHz). Analysis of NMR protons and NMR carbons begins by dissolving analytes (QLCs) using the solvent-deuterated methanol (CD3OD), as well as the comparison compound (quercetin). The solution made was added to the raw compound tetramethyl silane, (Si (CH 3) 4), and readings were carried out according to the specifications of proton NMR and carbon NMR devices. The spectra plotted by the JEOL show chemical shifts (δ) in parts per million (ppm) relative to the specified deuterated solvent. Spectra were calibrated using the residual solvent peaks for CD _3_OD (δH: 3.3 ppm; δC: 47 ppm) as appropriate. Coupling constants (J) were used quoted in Hz and were rounded to the nearest 0.1 Hz. Splitting patterns were observed abbreviated to singlet (s), doublet (d), triplet (t), quartet (q), and multiplet (m). ^23^^,^^29^^–^^31^

The molecular mass of QLCs was determined using ultra-performance liquid chromatography–mass spectrometry (UPLC-MS) connected to UNIFI software as a process control (Waters Corporation, 34 Maple Street, Milford, MA 01757, USA). The data were kept in the system computer and library of the instrument for matching the analytes. The column installed was octadecyl xylene (ODS) C18 stainless steel (Agilent product catalog number 5982-1111). The column oven and autosampler injectors were set at 40°C and 15°C and the volume capacity injector was set at 10 μL. The running parameters were adjusted along a gradient system using the mobile phase of A (acetonitrile pro chromatograph containing 0.1% formic acid p.a.) and B (water pro chromatograph containing 0.1% formic acid p.a). The flow rate was adjusted to 0.6 mL/min. The MS parameters were adjusted to the Tof MS ^E^ mode using electrospray ionization (ESI) at +/− and an acquisition range of 50–1200 Da. The following analysis criteria were adopted: mass error reading analyte ≤ 5 ppm; isotope match m/z root-mean-square (RMS) ≤ 6 ppm; and isotope match m/z RMS % ≤ 10 %, analyte intensity ≥ 300. For one fraction, the brake was < 4 in the fragment elucidation system match. ^32^^,^^33^

### Chicken kidney cell culture

Chicken kidney (CK) cells were cultured from healthy leghorn chickens (age, 3 weeks; approximately body weight, 0.8–1.0 kg). The chicken was free from specific chicken diseases caused by viruses, bacterial, and fungi as observed by a veterinarian in Surabaya-Indonesia (geographical coordinates 7° 20′ 09.7″ S, 112° 46′ 15.7″E, Surabaya-Indonesia). Animal Ethical Clearance was carried out through testing the animal ethics trial team from the Faculty of Veterinary Medicine, Universitas Airlangga, which had been proposed since the research began. The exam session consists of seven examiners, all of whom are veterinarians, and were specially appointed by the Faculty of Veterinary Medicine, Universitas Airlangga, to test the research proposal. The animal ethics clearance certificate was issued with No: 1.KEH.060.04.2023 on May 12 ^th^, 2023.

The cells were prepared, maintained, and propagated at the Research Center, Apply, Development of Veterinary Pharmacy Science, Faculty of Veterinary Medicine Universitas Airlangga. ^34^ The cells were developed and stimulated to grow in a 500 mL Roux plastic bottle in three types of medium: Eagle medium (EM), serum-free medium (SFM), and standard suspension medium (SSM). All cell culture media were sterile, free from pyrogenic substances, iso-tonic, iso-ionic, and iso-hydric.

Other environmental substances used in the *in vitro* test and cell culture were nutrient broth p.a, (Merck catalog number: MFCD01867738), *N*-acetyl ethyleneimine (AEI) 0.05% (Chemsky-Shanghai International Co., Ltd., catalog number: 32344), and sterilized phosphate-buffered saline (PBS) (Merck catalog number: P4474), Tween-40 (Merck catalog number: 9005-66-7), and polyethylene glycol (PEG) (Merck catalog number: 25322-68-3). Additional chemicals needed were 0.25% Trypsin Versen solution (Merck catalog number: 9002-07-7), sodium thiosulfate solution (Merck catalog number: 7772-98-7), citric acid (Merck catalog number: 77-92-9), trypan blue (Merck catalog number: 72-57-1), FBS (Merck catalog number: 12106C), and 10% Hibitane solution (Merck catalog number: 56-95-1). Antibiotics and fungicide were also used: Penicillin G w/v Oxoid Pharma 1.500.000 UI catalog number: CT0043B; Kanamycin Injection Meiji Pharma v/v 50 mg/mL), anti-fungi (Nystatin Taisho Pharma w/v 100.000 IU). We did not use commercial EM, SFM, and SSM media as they were not able to support the growth of CK cells in our labs. Thus, the formula of the CK culture cell medium was modified and optimized.

One liter of EM medium was supplemented with sodium chloride 6.4 g (Merck catalog number: 7647-14-5), potassium chloride 400 mg (Merck catalog number 7447-40-7), Mg _2_SO _4_.7H _2_O 200 mg (Merck catalog number 100034-99-8), glucose 4.5 g (Merck catalog number: 492-62-6), Fe (NO _3_) _3_.9H _2_O 0.1% 1 mL (Merck catalog number: 7782-61-8), aquabidest 750 mL (PT Kimia Farma Surabaya catalog number: 01 Aquadest), NaPO _2_H _2_.2H _2_O 140 mg (Anish Chemical-India catalog number: 28351090), and phenol red 0.1% 8 mL (Merck catalog number: 143-74-8). Other substances were antibiotics (Penicillin G w/v Oxoid Pharma 1.500.000 UI catalog number: CT0043B; Kanamycin Injection Meiji Pharma v/v 50 mg/mL), anti-fungi (Nystatin Taisho Pharma w/v 100.000 IU). The final ingredients of the formal SFM were sodium chloride solution 0.424 g (Merck catalog number: 7647-14-5) and sodium hydroxycarbonate 0.33 g (Merck catalog number: 15630-89-4). The composition of SSM was the same as SFM, with the addition of 10% of FBS.

### Preparation of primary chicken kidney cells

Cell culture was performed in a sterile biosafety cabinet (Laminar Air Flow Work Station, Sara Mechatronix) between 20°C and 23°C, as follows: one living organ was obtained and the surface membrane was cleaned and immediately inserted in PBS 25% (5–10 min) with gentle shaking to eliminate impurities. ^34^^,^^35^ The organ was directly inserted in the barrel of the 50-mL disposable syringe by first opening the plunger. Next, the plunger was pressed to pinch the organ and it then disintegrated into fine cells out of the syringe adapter part (without the presence of a needle) and was transferred into a centrifuge tube. The cells were gently washed with PBS and the tube was centrifuged at 3000× *g* for 10 min (Techne Genofuge 16M Lab Benchtop Centrifuge catalog number: 75004231). The cell pellet was collected, and the supernatant was discharged. An equal amount of EM was added and whisked to the cell pellet for 10 min with a rubber head glass dropper pipette to achieve a homogenous solution. This was followed by the addition of 5 mL of Trypsin Versen, and the mixture was immediately transferred into a Roux culture bottle and incubated for 35 min (37°C). Furthermore, the addition of 10% FBS while gently shaking was to inhibit the activity of Trypsin Versen, because the binding of serum with Trypsin Versen will inhibit the work of Trypsin. Finally, 250 mL of SSM was added to the bottle and incubated (Memmert ICO105 CO _2_ Incubator, adjusted use of 20% CO _2_) for 48 h at 37°C. Some of the solution and samples were tested for sterility by addition to the nutrient broth followed by incubation for 24 h at 37°C. Cell growth was examined every 24 h under an inverted microscope (Leica catalog number: DM IL LED) at 10×, 20×, and 40× magnification.

### Propagations of chicken kidney cells

After incubation for 48 h, the spent medium was removed and 25–30 mL of PBS was added. Then, 0.25% Trypsin Versene was added and incubated to dissociate the cells. After all cells had dissociated, fresh EM with 10% FBS and 3% PEG was added. Cell counting was performed with a hemocytometer (Fisher scientific catalog number: 22-600-107). Cell propagation was performed by repeating these three times to achieve stable growth. Cell passaging was performed to maintain cell growth for the antiviral assay.

### The sample size for the *in vitro* test

The sample size was calculated using Equation 1, with an assumed { *Z*_1_ − ( *α*/2)} at 1.96, with a significance of 0.05; *Z*_*β*_ at 1.645 by error limit of 5%; d at 3.62; Sa at 1.7; and Sb at 1.4. ^36^ As the N value was rounded to 5, the control and test groups were each to consist of five samples.

$$N=\frac{{\left[\left(Z_{1}-\frac{\alpha }{2}\right)+Z_{\beta }\right]}^{2}}{\frac{{\left(d\right)}^{2}}{{\left(S_{a}\right)}^{2}+{\left(S_{b}\right)}^{2}}}$$ (1)

### *In vitro* test quercetin-like compounds against to Newcastle disease virus

The experiment was performed at a temperature of 4°C–5°C. The first step was the preparation of the QLCs at 0.05% (w/v) in the EM vehiculum by making the preparation of QLCs into a suspension through the addition of 2% Tween 40 p.g. Furthermore, antibiotics and antifungals were added and then filtered using a 0.20-μm filter. After filtration, the solution was ready to be used.

First, the virus was diluted to 10 ^9^ by mixing 0.3 mL of virus substance plus 2.7 mL of EM. Then, 0.3 mL of the mixture was taken and put in vials plus 2.7-mL EM, with slow shaking. A similar process was performed for up to the ninth dilution. The antivirus test was started by taking 0.1 mL of each virus dilution in a 96-well microplate. Each dilution was repeated in five microplate wells by the determination of the sample size. In each well, 0.3 mL of 0.05% QLCs and 0.1 mL of EM contained FBS 10% and polyethylene glycol 3% were used.

The control wells consisted of three groups: one group as a negative control, free from virus, a positive control, with a virus at 10 ^−1^ dilution, and a bioactive control with virus at dilution 10 ^−1^ with AEI 0.05.

In the last stage, all stocks were as follows; 0.1 mL of all test materials ranging from dilute viral substances to QLC and CK cultures and all EM, as well as 10% FBS and PEG, were taken and dropped to a 10 mL tube containing thioglycolate broth (Merck catalog number: 116761) to test for contamination. Then incubation was performed in a CO _2_ incubator at 37°C for 24 h. Then, an assessment of the antivirus activity was performed by starting with the examination of all nutrient broth in the tube. If the tube test was free from contamination, the assessment continued. Antivirus assessment consisted of assessment of the positive control and negative control first followed by the examination of each well for a cytopathogenic effect (CPE) using an inverted microscope (Leica catalog number: DM IL LED) at 10×, 20×, and 40× magnification. ^15^ The examination technique for each microplate well and the CPE criteria are shown in Table 1.

**Table 1.** Technique and interpretation of the cytopathogenic effect using an inverted microscope.

Technique	Cytopathogenic effect funding	Interpretation
Magnification 10×, focusing well by working distance from the objective lens to top cover of the microtiter plate 40 mm, further enhanced 20× for 50 mm and 40× for 80 mm. In one well, observation was made following the letter S.	No formation in one cycle of the letter S.	Negative: Cell cultures continue to grow at the base of the microplate without being attacked by viruses.
	More than one formation in one cycle of the letter S.	Positive: Cell cultures are attacked by viruses so that the cells die and float on the surface.

### Statistical analysis

Statistical tests were performed using the probit analysis method for each virus dilution at which 50% antivirus activity was obtained for QLCs of 0.05% dilution, with a significance of α = 0.05. The statistical analysis was executed using the Statistical Package for the Social Sciences 24.0 software (IBM Corp., NY, USA).

## Results

The field virus isolate used in this study was identified as VO-expressed NDV with high levels of virulence ( Table 2 and Table 3). Virus identification and characterization of the NDV virus followed previous research on cases of duck outbreaks in the same area and using the same isolates. ^20^^,^^22^

**Table 2.** Profile the subject clinic.

No chicken	Virus-free	Commercial inactive vaccination				
	NV	AE	AI	ILT	IB	NDV
1 ^st^	Healthy	Immun	Immun	Immun	Immun	Immun
2 ^nd^	Healthy	Immun	Immun	Immun	Immun	Immun
3 ^rd^	Healthy	Immun	Immun	Immun	Immun	Immun
4 ^th^	Healthy	Immun	Immun	Immun	Immun	Immun
5 ^th^	Healthy	Immun	Immun	Immun	Immun	Immun

Note: NV = No vaccination, AE = vaccination of Avian Encephalitis, AI = vaccination of Avian Influenza, ILT = vaccination of Infectious Laryngotracheitis, IB = vaccination of Infectious Bronchitis, NDV = vaccination of Newcastle disease virus.

**Table 3.** Profile virulence of NDV in infected chickens 6 months age.

2nd-day post-vaccination administrated live virus of NDV, and each day was observed during the five days																													
NV					AE					AI					ILT					IB					NDV				
Days					Days					Days					Days					Days					Days				
1	2	3	4	5	1	2	3	4	5	1	2	3	4	5	1	2	3	4	5	1	2	3	4	5	1	2	3	4	5
+	+	+	†	†	+	+	+	+	+	+	+	+	+	+	+	+	+	+	+	+	+	+	+	+	-	-	-	-	-
+	+	+	†	†	+	+	+	+	+	+	+	+	+	+	+	+	+	+	+	+	+	+	+	+	-	-	-	-	-
+	+	+	+	†	+	+	+	+	+	+	+	+	+	+	+	+	+	+	+	+	+	+	+	+	-	-	-	-	-
+	+	+	+	†	+	+	+	+	+	+	+	+	+	+	+	+	+	+	+	+	+	+	+	+	-	-	-	-	-
+	+	+	†	†	+	+	+	+	+	+	+	+	+	+	+	+	+	+	+	+	+	+	+	+	-	-	-	-	-

Note: NV = No vaccination, AE = vaccination of Avian Encephalitis, AI = vaccination of Avian Influenza, ILT = vaccination of Infectious Laryngotracheitis, IB = vaccination of Infectious Bronchitis, NDV = vaccination of Newcastle disease virus, + = Infection of the NDV, − = Not infection of NDV, † = Died chicken.

The NV group was seen appearing in every individual chicken with symptoms of NDV virus infection, like a symptoms as follows; the head was bowed, there was discharge in the mouth and nose and body heat increased to an average of 41°C. Typical symptoms are that the chicken often turns its head and on lung auscultation examination, secretory noises can be heard in the lungs. These symptoms were not found in the group of chickens that had been immunized against active NDV.

**Figure 1.**
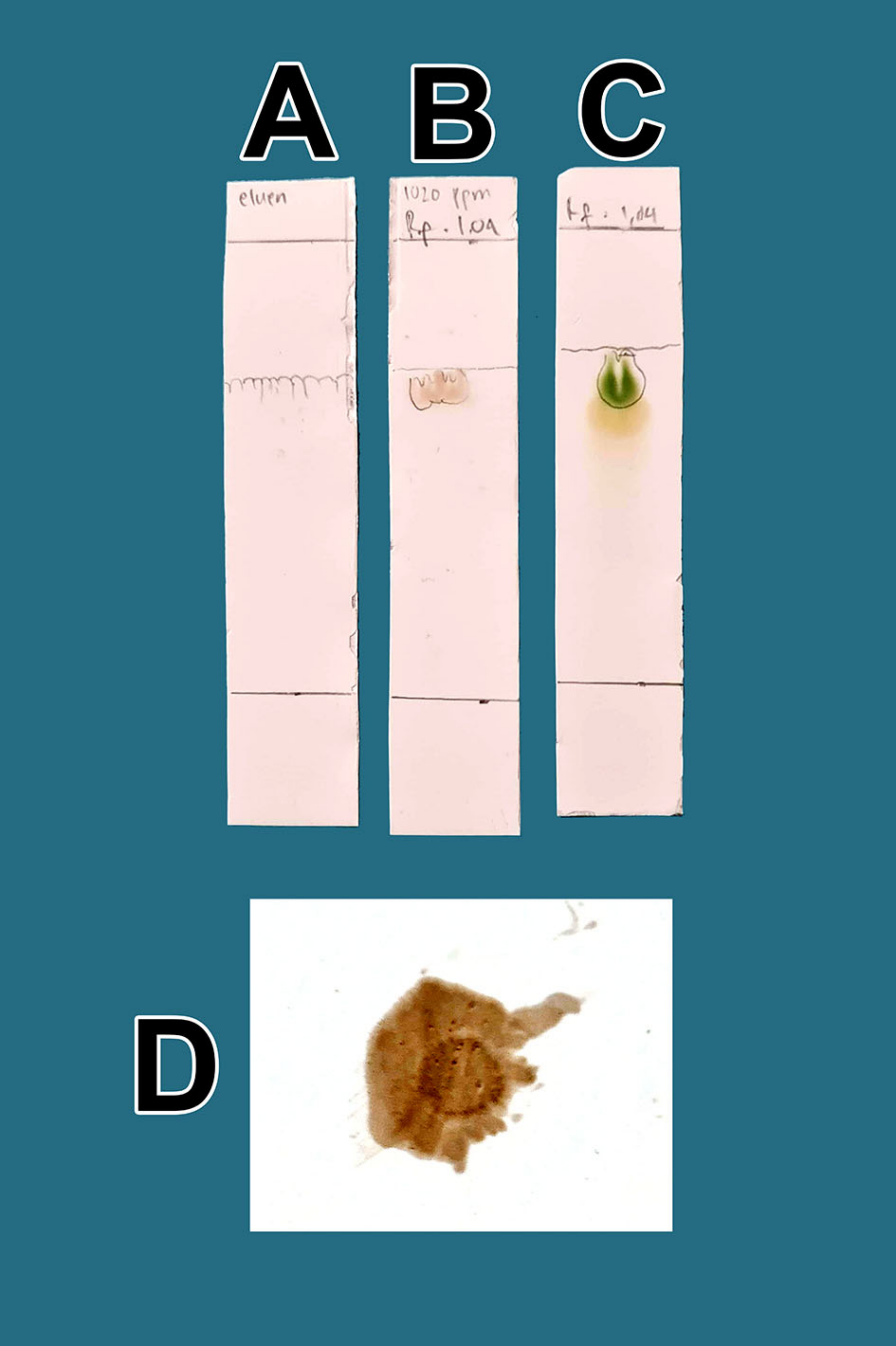
Screening flavonoids via thin-layer chromatography and observed under 366 nm UV radiation. A. Control eluent, t mobile phase, B. Flavonoid. C. Standard quercetin. D. Flavonoid sprayed with ammonium solution giving a red color.

From a total of 5 kg BD leaves, approximately 5 g of flavonoid raffinates were obtained. For all raffinates, flavonoids were present as determined by both methods tested. These samples were known to contain flavonoids because of the change in color to green following the addition of a small amount of Mg into solutions with the raffinates and HCl. This was further tested by determining the R _f_ value of the chromatography thin-layer elution results and comparing it to that of the standard; the R _f_ value of QLCs (102 ppm, B) was equivalent to the R _f_ standard (100 ppm, C) as shown in Figure 1. The eluent mobile phase as a control appeared on the TLC marked as A. After elution, the substance spots were determined to be flavonoids after spraying with an ammonium solution revealed a red color (D).

**Figure 2.**
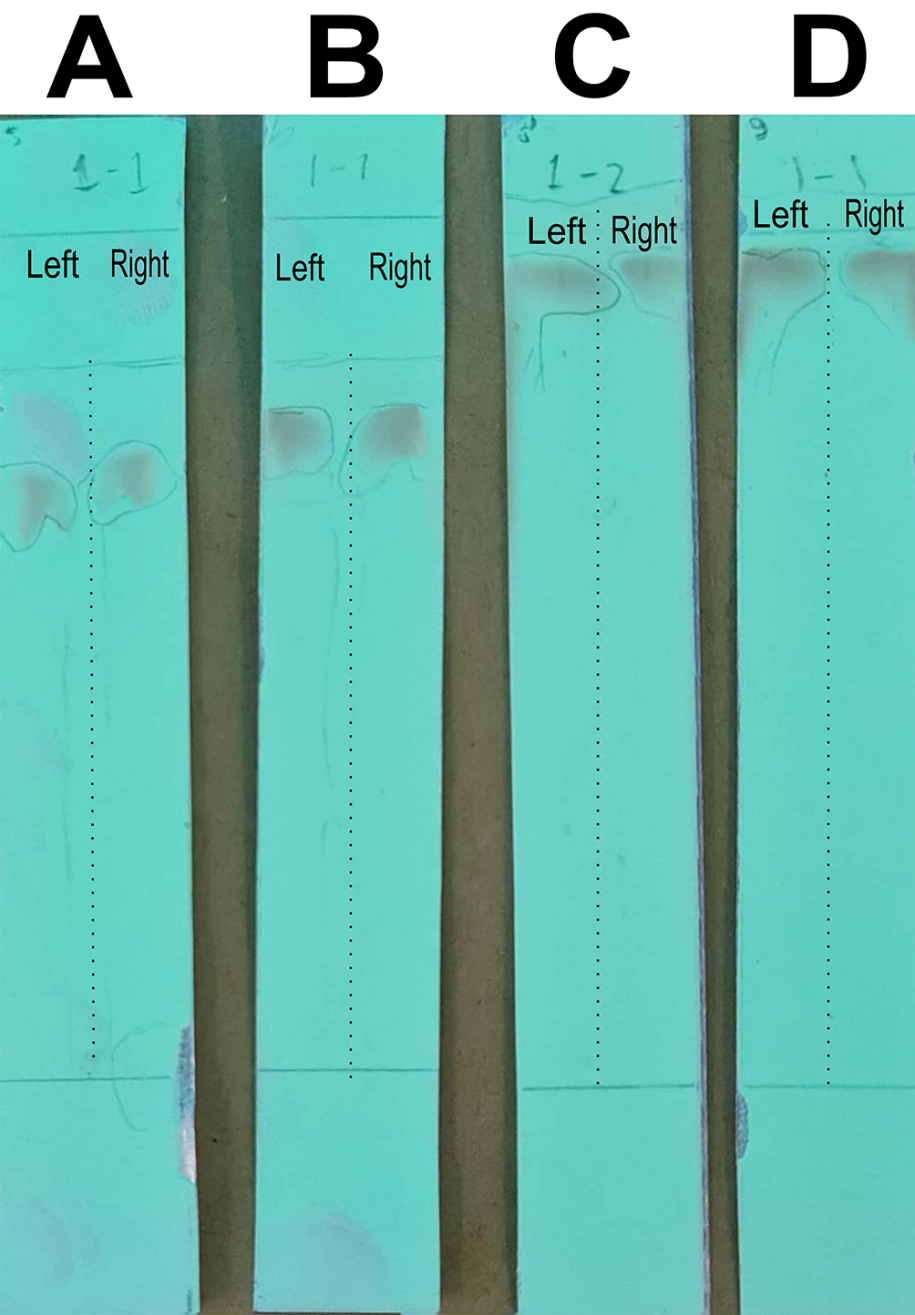
Elution of quercetin-like compounds (QLCs) in thin-layer chromatograph (TLC) by mobile phase of water pro chromatograph–methanol pro chromatograph at 4:6 (A); 3:7 (B); 2:8 (C); 1:9 (D). Each TLC strip contained two compounds: (left) samples and (right) standard of quercetin p.a.

The analysis of the mobile phase fraction between water–methanol and the stationary phase in TLC is shown in Figure 2. Figure 2(A) shows that the adsorption–partition power against flavonoids was strong. TLC Figure 2(A); the left image shows QLCs and the right image shows the standards; R _f_ was 1.02. Figure 2(B), using a 3:7 phase fraction of mobile air–methanol shows the adsorption–partition power of QLCs (left) compared with the standard (right) moderate. The fraction of 3:7 was suitable for applications in HPLC (R _f_ 1). Figure 2(C), using the mobile air–methanol phase fraction of 2:8, shows that the adsorption–partition power of QLCs (left) compared with the standard (right) is weak and therefore unsuitable for applications in HPLC (R _f_ = 0.98). Spot QLCs are longer than Figures 2(A) and 2(B). Figure 2(D), the mobile fraction water–methanol at 1:9 shows that the adsorption–partition power of QLCs (left) compared to the standard (right) is very weak; the adsorbed partition spot becomes longer and is not suitable for applications in HPLC (R _f_ = 0.986).

**Figure 3.**
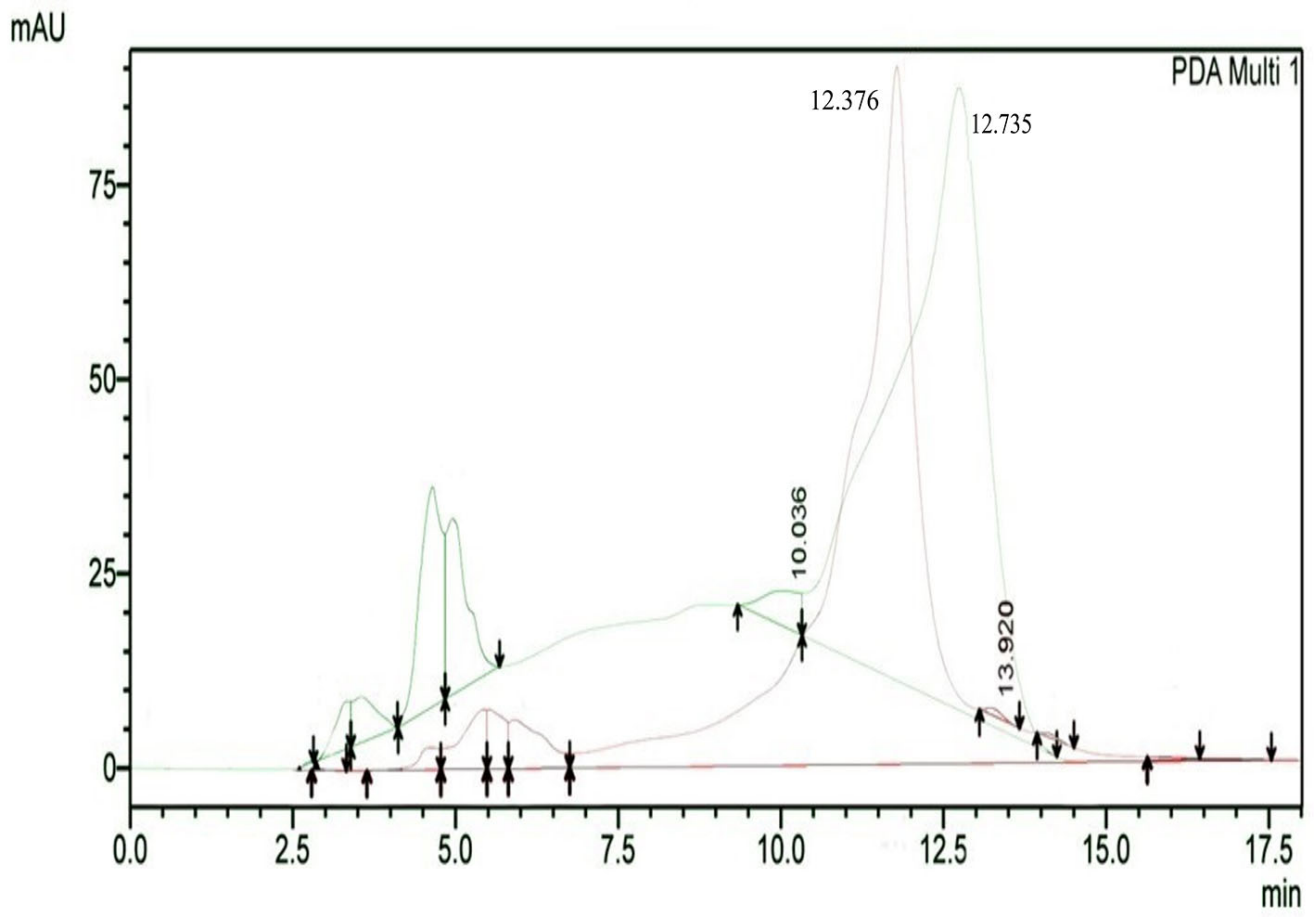
The overlay of chromatograms of quercetin-like compounds in magenta with a peak at a retention time (RT) of 12.376 min and the standard compound in green with a peak at a RT of 12.735 min.

The results of HPLC RT in Figure 3 show the peaks of the analyte (QLCs) in magenta and the standard in green color ( Figure 3). The peak RTs of the two chromatograms were similar. The tolerance range between the retention times of analytes and the RT was estimated to be <5 min.

**Figure 4.**
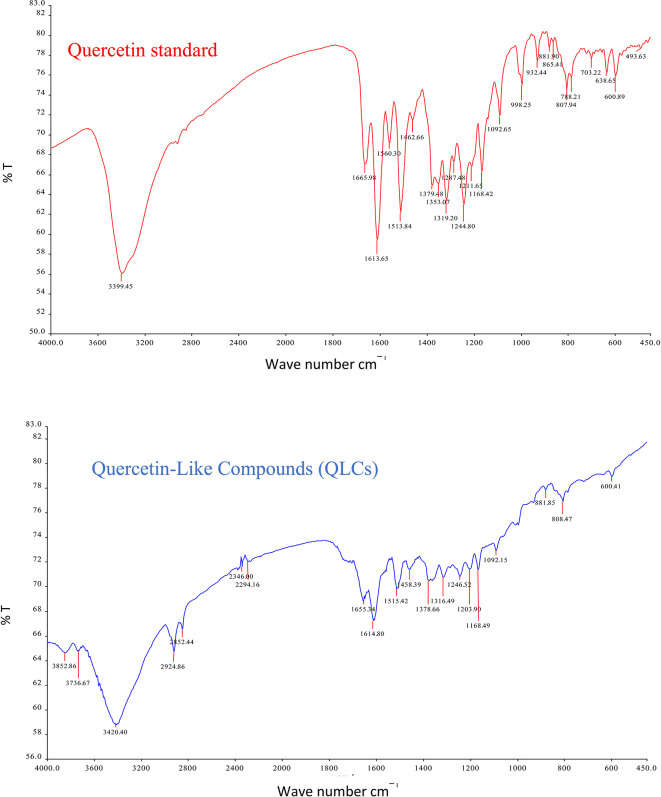
Infrared spectra of quercetin standard (red) and quercetin-like compounds (blue).

The IR spectrum of the standard and QLCs are shown in red and blue, respectively, in Figure 4. It is generally observed that the standard spectral intensity (%T) is greater in QLCs ranging from wave numbers of 4000 cm ^−1^ to 400 cm ^−1^. However, there is still a higher intensity of QLCs than the standard, especially for wave numbers of 2925 cm ^−1^ to 2853 cm ^−1^. However, in the fingerprint region, at wave numbers of 1200 cm ^−1^ to 700 cm ^−1^, there is no high spectral intensity. In general, Figure 4 shows that there are some similarities in the infrared spectra of the standard and the QLCs. The element water always appears at wave numbers from 3600 cm ^−1^ to 3000 cm ^−1^, and this shows that the two compounds are polar and soluble in organic solvents. The specificity of the QLCs IR spectrum compared to the standard shows that there are peaks of the spectrum in the region before the fingerprint region and in the fingerprint region, although it has a %T value not too high. The IR spectra peaks for the analytes and standard that indicate similar compounds were the regions of 3420.40 cm ^−1^ and 3399.45 cm ^−1^ for the OH ^−^ ion; 1655.34 cm ^−1^ and 1669.98 cm ^−1^ assigned to the C=C ring, or 2- or 3-band stretching vibrations. The peaks at 1655.34 cm ^−1^ and 1614.80 cm ^−1^ for the analyte and 1665.98 cm ^−1^ and 1613.65 cm ^−1^ for the standard were assigned to the C=O bond. The other peak of 1515.42 cm ^−1^ for the analyte and 1513.84 cm ^−1^ for the standard were assigned as a C=C stretching. The peaks at 1458.39 cm ^−1^ and 1378.66 cm ^−1^ for the analyte and 1462.66 cm ^−1^ and 1379.48 cm ^−1^ for the standard were assigned to the –O–H bending of aromatic compounds. The peaks at 1316.49 cm ^−1^ and 1246.52 cm ^−1^ for the analyte and 1353.07 cm ^−1^, 1319.20 cm ^−1^, and 1244.80 cm ^−1^ for the standard were assigned to an aromatic phenol ring. Other peaks, at 881.85 cm ^−1^ and 808.47 cm ^−1^ in the analyte and 881.90 cm ^−1^ and 807.94 cm ^−1^ in the standard, were assigned to a phenol –C=C– bond. The fingerprint area of analytes at 600.41 cm ^−1^ and of the standard at 600.89 cm ^−1^ indicated the same specific functional compounds of an aromatic or phenolic –COO– group.

**Figure 5.**
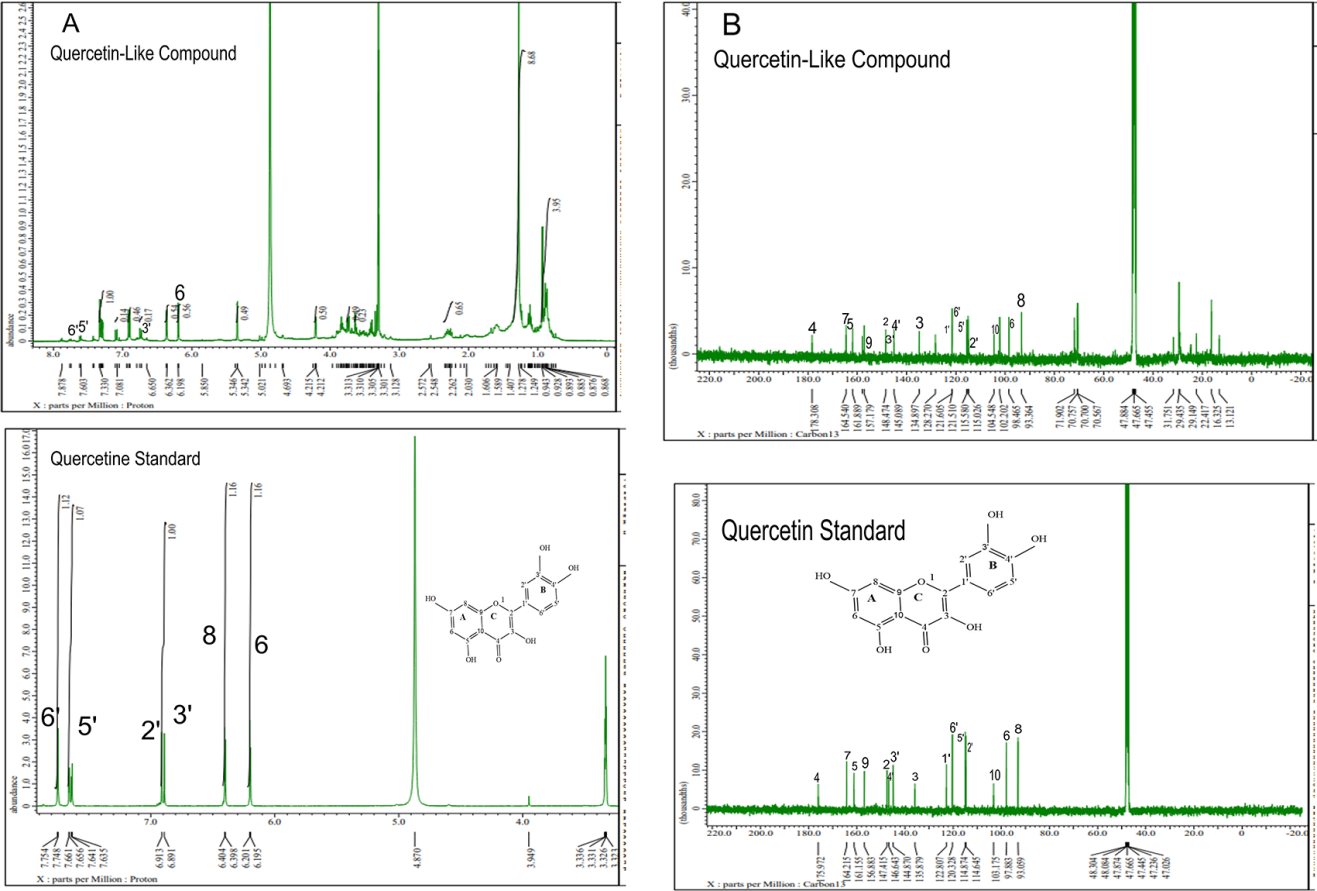
Spectrum A: Proton nuclear magnetic resonance ( ^1^H NMR) of quercetin-like compounds (QLCs) the analyte and quercetin pro analysis as the standard dissolved in deuterated methanol. Spectrum B: Carbon nuclear magnetic resonance ( ^13^C NMR) of QLCs as the analyte and quercetin pro analysis as the standard dissolved in deuterated methanol.

The analysis of ^1^H NMR and ^13^C NMR is presented in Figure 5(A) and 5(B), respectively; in each part, the upper part shows the QLCs and the lower part the standard. From Figure 5(A), the proton spectrum of QLCs can be identified, namely the chemical shift (δ) at 4.800 ppm is CD _3_OD, while the chemical shift (δ) CD _3_OD of the standard quercetin spectrum is 4.870 ppm. Figure 5(B) shows the chemical shift (δ) position of the CD _3_OD in ^13^C NMR of QLCs and quercetin standards occurred at 47.665 ppm.

**Figure 6.**
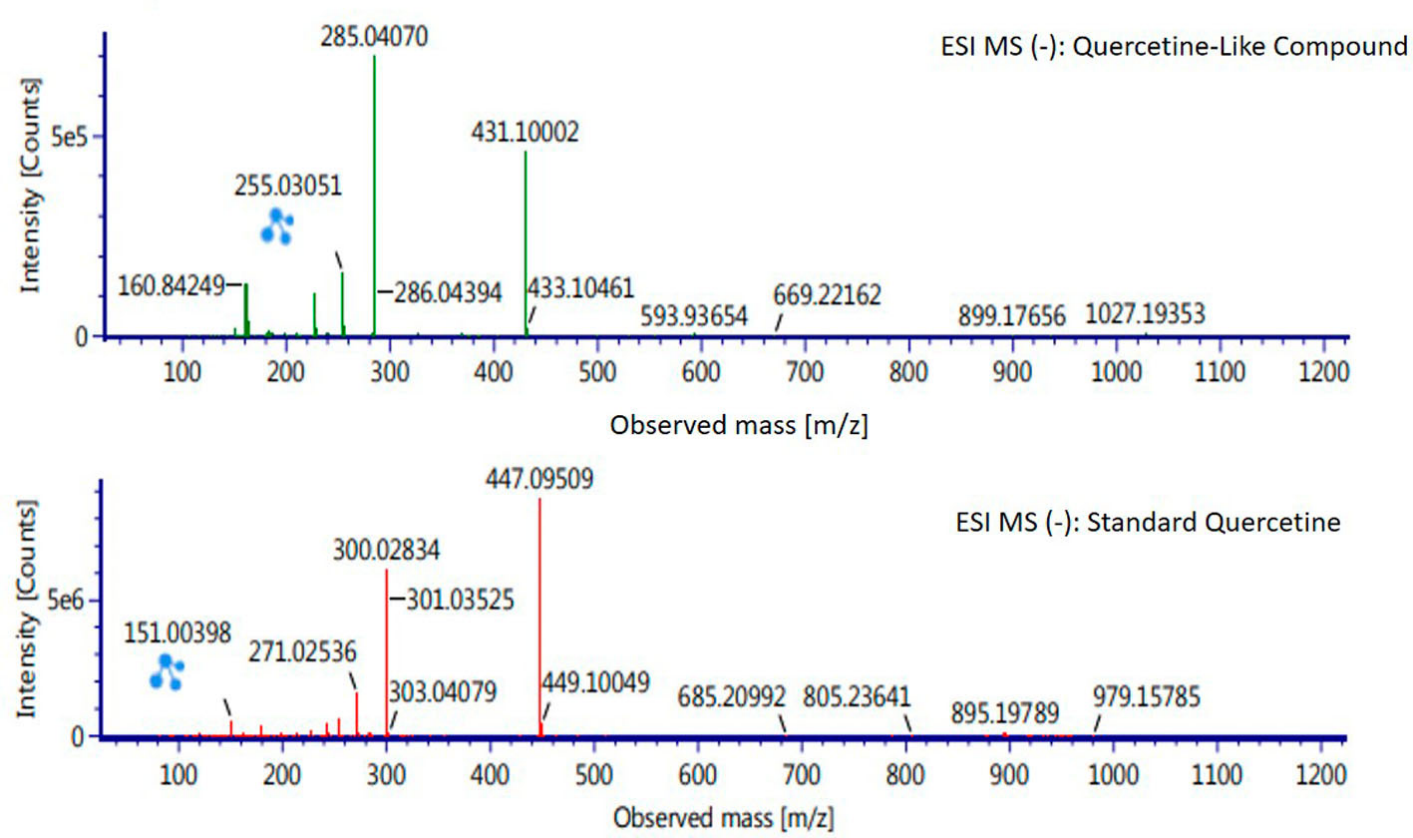
Mass spectrum by electrospray ionization of quercetin-like compound and quercetin standard dissolved in methanol pro chromatograph at 1 ppm.

Analysis of BD plant QLCs and identification was performed based on the retention time for quercetin standards; five bioactive were identified as positively or negatively ionized molecular fractions (m/z). However, not all were identical when further traced to the amount of carbon, hydrogen and oxygen atoms bound to the aromatic rings of polyphenols. The results of the m/z (+) molecular mass-based analysis showed that QLCs have polyphenol elements similar to genistein, whereas m/z (−) results were similar to bioactive 5,7,8,3′,4′-pentamethoxyflavonone, 7-hydroxy-1-methoxy-2-methoxyxanthone, and pelargonidin 3-glucoside. Table 4 shows the molecular mass of the five bioactive compounds. Of the four bioactive compounds outside the standards, only one of had similarities with one of the standard mass molecular fragments, namely hydroxy-1-methoxy-2-methoxyxanthone. Further similarities between bioactive and hydroxy-1-methoxy-2-methoxyxanthone are shown in Figure 6.

**Table 4.** Analysis of molecular mass of quercetin-like compounds compared with quercetin standard.

Formula	Observed retention time (min)	Total fragments found	Isotope match m/z root-mean-square (ppm)	Isotope match intensity root-mean-square (%)	Response	Adducts
Genistein	11.20	29	1.48	3.42	15.635	+H
5,7,8,3′,4′- Pentamethoxyflavonone	10.71	5	2.94	9.29	204	−H
7-Hydroxy-1-methoxy-2-methoxyxanthone	11.29	8	1.78	9.4	1.890	−H
Pelargonidin 3-glucoside	11.29	5	3.48	6.14	753	−H
Quercetin standard	12.58	14	1.79	3.27	24.0734	+H
	10.16	26	2.91	0.86	17.224	−H

**Figure 7.**
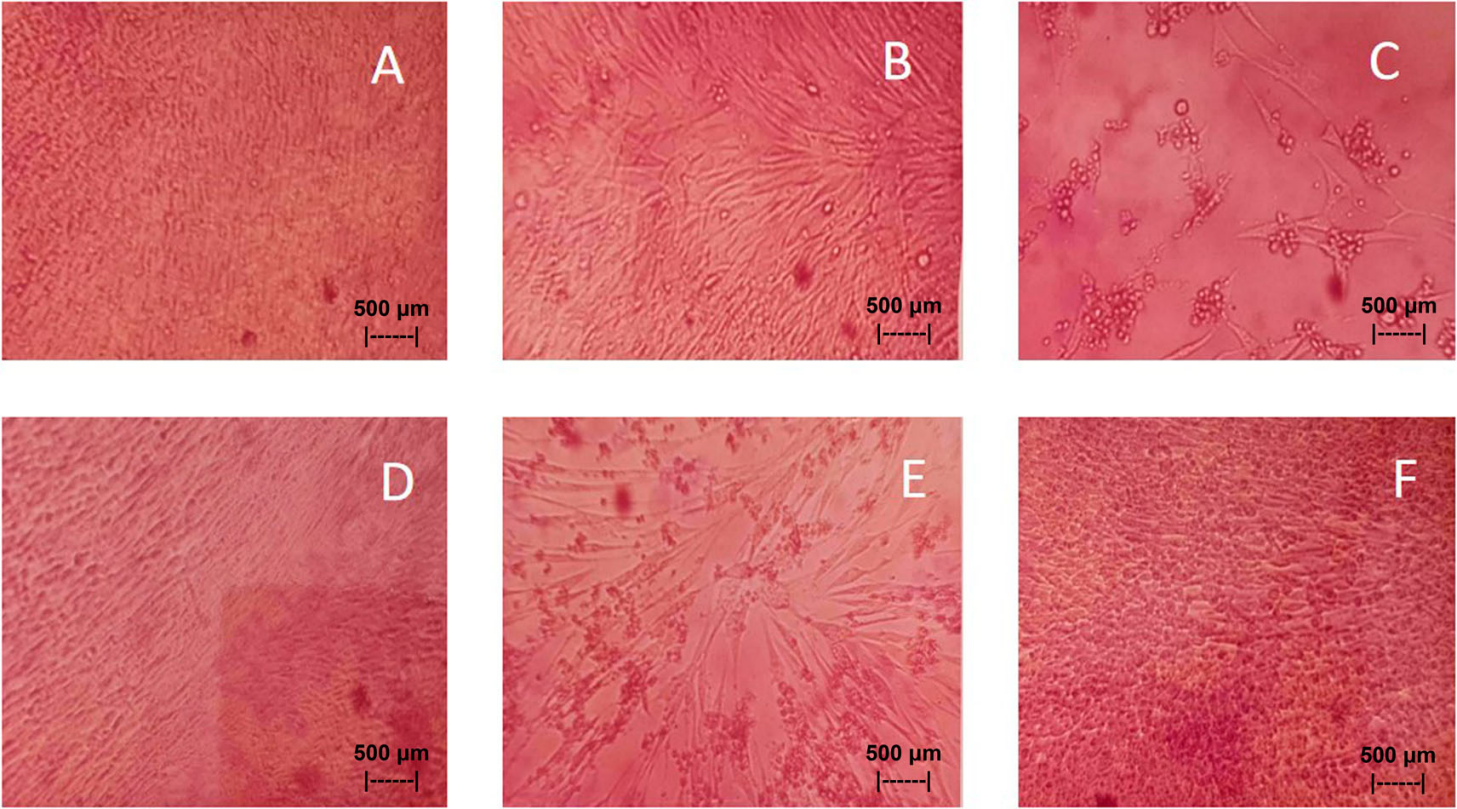
(A) *In vitro* test of 0.05% quercetin-like compounds (QLCs) against of live Newcastle diseases virus (NDV) at a dilution of 10 ^−5^ in a microplate. (B) *In vitro* test of 0.05% QLCs against live NDV at a dilution of 10 ^−4^. (C) *In vitro* test of 0.05% QLCs against live NDV at a dilution of 10 ^−1^. (D) Negative control of chicken kidney (CK) cell culture in Eagle’s medium grown on microplate wells. (E) Positive control of CK cell culture containing of live NDV at a dilution of 10 ^−1^. (F) Bioactive control of CK cell culture containing 0.05% acetyl ethylene imine and live NDV at a dilution of 10 ^−1^ (Normal image brightness at magnification 100×).

Results of the *in vitro* test of QLCs against NDV, after incubation for 24 h showed the nutrient broth in the control tube, was free from contamination. The observations at serial virus dilutions of 10 ^−1^ to 10 ^−9^ compared with the positive controls and negative controls are shown in Table 5 and Figure 7. Probit analysis of the 50% endpoint with a value of 10 ^−2.314^ showed that diluting the virulence of the NDVs was able to reduce its ability by half by observing CPE findings in CK culture ( Figure 6, p<0.05).

**Table 5.** *In vitro* test of antivirus activity of 0.05% quercetin-like compounds against Newcastle disease virus compared with acetyl ethylene imine 0.05%.

Control			Dilution of Newcastle disease virus								
Negative	Positive	0.05% AEI	10 ^−1^	10 ^−2^	10 ^−3^	10 ^−4^	10 ^−5^	10 ^−6^	10 ^−7^	10 ^−8^	10 ^−9^
−	+	−	+	+	+	+	−	−	−	−	−
−	+	−	+	+	+	−	−	−	−	−	−
−	+	−	+	+	−	−	−	−	−	−	−
−	+	−	+	+	−	−	−	−	−	−	−
−	+	−	+	−	−	−	−	−	−	−	−

Note: − Chicken kidney cells had no CPE; + Chicken kidney cells had CPE.

## Discussion

The virus used and declared by VO is the result of an examination carried out by VO. It was also stated that NDV field isolates have virulence capable of causing 60% mortality of infected chickens aged 6 months. The method of virulence testing by VO is carried out using biological observations. These results show that the longer the chicken is infected with NDV, the risk of death will increase the risk of death for infected chickens. ^37^ The average ability to survive infection with artificial infections of the NDV virus only lasted three days out of the five-day observation plan. ^38^ This fact is different when compared to other chickens that have been vaccinated with the vaccines AE, AI, ILT, and IB. Survival ability for AE, AI, ILT, and IB groups where the group has NDV infection, there may be cross immunity that can indirectly protect the chicken’s body infected against the NDV virus. This can happen when immunity to AE, AI, ILT, and IB has emerged.

Overview of field test methods with the latest technology, until now has not been developed much. But later it will be able to be done using artificial intelligence technology based on science data that can be collected from various countries. The principle of the test is based on a mathematical model involving many factors that can affect the results of the challenge test. Currently, there are several types of field tests based on artificial intelligence, one of which is the type of vaccine used in COVID-19 cases. ^39^

The separation of bioactive components to obtain QLCs from the crude methanol extract of BD leaves is easy in principle, but the yield is very small. For example, 5 kg of BD leaves yields approximately 250 mg of QLCs (0.005%). The low yield of QLCs obtained indicates that sample preparation must be carefully performed and indicates a potential loss of analytes during the separation and refining processes. One of the precautionary steps in the framework of laboratory work processes related to minimizing the loss of analytes is the identification of medicinal plants performed by an institution/authority. QLCs results are generally also obtained when the same isolation is carried out on secondary metabolites from other mistletoe leaves, with an average of 1 to 0.005%, especially mistletoe plant species. ^25^^,^^33^ BD plants contain different active ingredients, and one of the areas where plants have the highest active ingredient content is the sampling location for this study (Muara Enim District, south of Sumatera, Indonesia).

The quality of bioactive content is different between areas owing to differences in nutrients, solar radiation received, and air quality. Enhancing these factors results in higher bioactive content in mistletoe. In Figure 1(B), the quality of flavonoids containing QLCs has R _f_ (1.04 cm), equivalent to quercetin (point C, 1.64 cm); the R _f_ is only 0.60 cm different. Spraying with ammonium produces a red color [ Figure 1(D)]. These findings suggest that the separation of flavonoids from the crude extract of BD leaves has occurred correctly.

These results show that water–methanol fractions of 2:8 and also 1:9 was not satisfactory in separating the QLCs from other impurities. Thus, a fraction of 3:7 was selected as optimal. The most difficult part to separate is the components that contain halide elements that bind to non-halide elements and are toxic. To overcome this, it must be ensured that when withdrawing flavonoid elements, no elements are found that can cause organ damage through toxic elements in the extract. This has been confirmed for BD plants in previous studies. ^15^^,^^40^

Bioactive QLCs appear in the chromatogram at RT of 12.376 min. The peak in the standard chromatogram occurred at RT of 12.736 min. Thus, QLCs will be found in retentate collected between 12.00 min to 13.00 min. Figure 3 shows that between the RTs of 12.00 min to 13.00 min, there are no impurities in the chromatogram peaks, so it is believed that those compounds collected in this RT range are QLCs. In this study, monitoring the peak appearance of the QLC chromatogram was carried out using three sample injections and one standard technique. The purpose of the technique was to monitor possible shifts in retention time for analytes. This was done considering that the peak area of standard natural ingredients such as quercetin resulting from HPLC analysis generally has more than one peak area. This was necessary because of the phenomenon of frequent shifting in the appearance of analyte and standard chromatograms when using HPLC. If the shift in the RT chromatogram lasts between 2–3 minutes, this can be tolerated. However, if it is >5 min, the column must be cleaned with the mobile phase so that the stationary column retains a high affinity for binding QLCs. From the standard chromatogram in Figure 3, the green graphic line shows that at RT 10.036 minutes other elements were found besides the main element (polyphenols), it is suspected that these elements were 2-hydroxy groups facing each other in the ortho molecular position of the aromatic ring. This structure is not found in QLCs, but if the analytes remain in the mobile phase solvent too long, then it is possible that the hydroxyl bond will break causing a peak to appear. To avoid the instability of the analytes, the time range for dissolving analytes in the eluent mobile phase is limited to 8 h.

FT-IR analysis is intended to identify the similarity of functional groups through the fingerprint region, as shown by comparing the QLCs (blue spectrum) and quercetin (red spectrum) between 600 cm ^−1^ and 400 cm ^−1^ ( Figure 4). However, QLC wave numbers in non-specific areas, namely between wave numbers 1800 cm ^−1^ to 600 cm ^−1^, can also be used as a study of the bioactive characteristics to be investigated. There are two things about FT-IR analysis, there was an IR spectrum with similar wave numbers between QLCs and standards, but there was also an IR spectrum at certain wave numbers that do not match QLCs with standards. The mismatch of the IR spectral between QLCs and standards was well understood considering that bioactive withdrawals in natural plant preparations were difficult to draw the purity of active ingredients from disruptive elements. The mismatch of the IR spectrum reaches up to 40% and this was often found in studies using plants as objects of study. ^41^^,^^42^

Analysis of proton ( ^1^H) and carbon ( ^13^C) NMR of standard and analytes was performed using a 99.9% (w/v; 0.36 M) solution standard of quercetin and analytes dissolved in methanol-d _4_ were acquired using the JEOL JNM ECS-400 NMR spectrometer ( Figure 5). This spectrum reveals three types of proton and carbon signals as follows: aromatic protons carbon and hydroxy protons carbon of the analyte and standard, and solvent proton carbon. The main structure of a QLC is a benzopyrone with three hydroxyl group substitutions (positions 3, 5, and 7) and at proton ( ^1^H NMR) positions of H-6 δ 6.198 ppm and H-8 δ 6.401 ppm. Other signals for the 2′,5′, and 6′ B ring of the quercetin structure were protonated at δ 6.902 ppm, δ 7.648 ppm, and δ 7. 751 ppm. The carbon NMR ( ^13^C NMR) 8, 10 of QLCs were present on the A ring of quercetin at δ 93.059 ppm and the AC ring of quercetin at δ 103.175 ppm. Other atoms of QLCs were present on the C ring of quercetin as follows: 2C δ 147.415 ppm, 3C δ 135.879 ppm, 4C δ 175.972 ppm, and 7C δ 164.215 ppm on the A ring of quercetin, and 9C δ 156.883 ppm on the AC ring of quercetin.

The results of the RT analysis of QLC molecules show that the similarity of RT to standards cannot be used as the main criterion. The combination of observations of the number of atoms, retention time, and the similarity of mass is required. In Table 4, it is shown that the mass (Da) of 5,7,8,3′,4′-pentamethoxyflavonone, and pelargonidin 3-glucoside is between 372–375 Da and 468–470 Da. The molecule in that period is not equivalent to the standard, which is between 302–303 Da. Thus, the two bioactive compounds are not very similar to QLCs. In terms of the molecular formula of the two bioactives, namely C _20_H _22_O _7_ for 5,7,8,3′,4′-pentamethoxyflavonone and C _21_H _21_ClO _10_ for pelargonidin 3-glucoside, they are not similar to the quercetin standard (C _15_H _10_O _7_). In contrast, genistein (C _21_H _20_O _10_) has a molecular mass of 432–434 Da, not very similar to the standard and is not included in the components forming QLCs. Thus, the compound that bears a resemblance to quercetin is 7-hydroxy-1-methoxy-2-methoxyxanthone and has a molecular structure of C _15_H _10_O _6_ as shown in Figure 6, at Electrospray Ionization-Mass Spectrometry (ESI-MS) (m/z) 431.10002, not too far from quercetin i.e., ESI-MS (m/z) 447.09509. Molecular ion fractions also found in (m/z) QLCs ESI-MS (−) 255.03051 show their proximity to molecular ion fractions (m/z) of standard ESI-MS (−) at 271.02536. The difference in ESI-MS (−) 15.99479 between the two can be understood by possible binding with other elements. Another molecular ion fraction is ESI-MS (m/z) 899.17656 for QLCs compared to ESI-MS (m/z) standard 185.19789 for standards, also bearing similarities. ^43^

The results of *in vitro* studies show that the administration of a suspension of 0.05% QLCs can inhibit the NDV growth starting from 10 ^−2^ dilution, as shown in Table 5. Thus, the more diluted the virus, the more potent the QLCs suspension. Using probit analysis, the virulence ability of NDV following QLC administration of up to 25% will occur at dilution 10 ^−2.937^. The ability to reduce the virulence of NDV to zero by administration of 0.05% of QLCs was found for a dilution of 4.462. When considering the active structure and quercetin analogy, hydroxyl elements that are bound to a single aromatic element such as phenol, and located opposite each other to the ortho position have the ability to kill the NDV. It is known that the arrangement of the base pair (bp) of the NDV virus (rNDVs) is as follows: 5′AGCTTTGTTTAAACTTAGAAAAAATACGGGTAGAAGGCCACCATGGGCT130 GCCTGGGCAAC3′. ^44^ The bp rNDVs have the ability to infect eukaryote aerobic cells, so they can easily attack the upper respiratory system and then infect the nerve cells of the brain. ^45^ When associated with the molecular structure of QLCs, the hydroxy groups will bind to the bp part in the adenine guanine and tyrosine regions. The xanthone group contained in QLCs will also bind to adenine–quinine–tyrosine, thus inhibiting the process of viral replication in host eukaryotic cells without killing host cells. ^46^ In such circumstances, the virus will gradually die itself. Xanthone derivatives will also bind to other bp such as cytosine with the effect of disrupting viral replication without causing death in host cells. Hydroxyl bonds and xanthone derivatives have a high affinity for base pairs that require high energy such as viral replication activities. ^47^ The binding affinity will not target the base pairs of the host cell considering that the energy strength of viral replication is higher than the physiological activities of the host cell. The virucidal activity of QLCs is also influenced by the lipophilic nature of the BD extract compounds; they can easily penetrate the surface of the lipid bilayer of host eukaryotic cells. Thus, the QLC components kill the virus more quickly; this phenomenon is shown in Figures 7(B) and 7(C). The ability to utilize host cell organelles in NDVs is very high and this is needed for viral replication. ^48^ In such circumstances, many host cells will die as seen in Figure 7(E) for the positive control and can be compared to the negative control 7(D). Replication behaviour can be stopped with 0.05% AEI, and it can be seen in Figure 7(F) that CK cells are still growing, although are not very healthy with blackish elements in the medium. The black appearance of the medium in Figure 7(F) is clearly distinguished from control cells where the medium is dark red [ Figure 7(A)].

The element QLCs in the form of 7-hydroxy-1-methoxy-2-methoxyxanthone are ligands with a phenolic part bound to a strong electronegative group that can resonate and interact with the substrate elements of viral base pairs that have a high-energy metabolism. At the time of the complexion, the strong electronegative binding of the ligand to the viral substrate was very strong. This property causes the ability of the virus to replicate very limited, resulting in the phenomenon of virucide.

The phenomenon of NDV viricide in 0.05% QLC suspension is thought to be suitable for use against other types of aerobic viruses such as foot and mouth diseases in cattle or lymph skin diseases in cattle, buffalo, sheep and goats. It is possible that it can be used against viral infections in humans. ^49^ The virucidal potential of QLCs has good prospects, although an increase above a concentration of 0.05% does not necessarily result in higher virucidal power. This is due to the influence of the host’s base pair, which is at risk of disrupting the host cell metabolic cycle resulting in the death of the host cell. There are distinct advantages when using QLCs as a virucidal bioactive, namely the natural properties of these QLCs make them safe for the host body and do not carry the same risks as residues of veterinary drugs. ^50^

The research results that have been found, compared with other researchers even from different plant medicines, show that the ability of the secondary metabolites found is one and a half times to two times stronger as an anti-virus. ^2^^,^^5^^,^^17^^,^^19^ The suggestion in this study is that it is necessary to conduct similar research using sprouted chicken egg media as an object of observational infection. Thus, it will get the infection value from the sample used. ^51^^,^^52^

## Conclusions

The bioactivity of QLCs arises from the phenolic elements with the addition of hydroxyl groups and the aromatic structure of xanthone. These two elements can interact with viral rRNA base pairs. Thus, QLCs extracted from BD leaves are capable of exerting virucidal power against NDVs to a concentration of 0.05% (p<0.05). The virucidal power of 50 end points for QLCs against NDV was found with a dilution of 10 ^−2.314^. The weakening of NDV infection to 25% after administration of 0.05% QLCs occurred with a dilution of 10 ^−2.937^. The NDVs were eliminated completely after administration of 0.05% QLCs at a virus substances dilution of 10 ^−4.462^. The virucidal power does not interfere with the host cell’s metabolic processes; the host cell continues to grow and develop. Thus, bioactive QLCs are potential virucidal active compounds for both humans and animals. We hope that in the future they may be successfully developed into safe veterinary drug compounds.

## Data Availability

Figshare: *In vitro* analysis of quercetin-like compounds from Mistletoe Dendrophthoe pentandra (L.) Miq as a potential antiviral agent for Newcastle disease. https://doi.org/10.6084/m9.figshare.22587166. ^53^ This project contains the following underlying data:
•
*In Vitro* Test-Edit-1.spv (Output of probit analysis test groups) • Data of *In Vitro* Test-Edit-1.sav (Probit analysis data by cytopathogenic effect observation in microtiter plate, groups test vs. groups controls positive & control negative) • FT-IR Edit-1.pdf (Overlay the specific spectrum of Quercetin-Like Compounds vs Quercetin in wavelength number 400 cm ^-1^ to 400 cm ^-1^) • Certificate English editing.pdf (Certificate from ENAGO Corp., for editing grammar and sentences). • LC-ESI-MS.pdf (Analysis of Quercetin standard using LC-ESI-MS) • LC-ESI-MS.pdf (Analysis of Analytes as Quercetin-Like Compounds using LC-ESI-MS) • Data set *in vitro* test. pdf (An observed of cytopathogenic effect on the microtiter plates growth up well of virus NCD in chick kidney cells and add the analytes as Quercetin-Like Compounds) • Data statistic.pdf ((Dose-response as cytopathogenic effect of cells culture after add the dose dilution virus 1/10 to 1/100.000) • Nuclear Magnetic Resonance carbon of Analytes.pdf (Carbon NMR spectrum of analytes as an extract benalu duku) • Nuclear Magnetic Resonance proton of Analytes.pdf (Proton NMR spectrum of analytes as an extract benalu duku) • Nuclear Magnetic Resonance carbon of Standard Quercetin.pdf (Carbon NMR spectrum of standard as a quercetin) • Magnetic Resonance proton of Standard quercetin.pdf (Proton NMR spectrum of standard as a quercetin) • Spectrum FT-IR standard Quercetin and raw material Quercetin-Like Compounds.pdf (Spectrum Quercetin and Quercetin-Like Compounds at 4000 cm ^-1^ to 450 cm ^-1^) • Overlay peak area of HPLC.pdf (peak area of Quercetin vs. Quercetin-Like Compounds). • Screening flavonoid via Thin Layer Chromatograph.pdf (available of Quercetin-Like Compounds in flavonol of extract benalu duku. •
*In vitro* test virus of NCD in chick kidney cell vs Quercetin-Like Compounds. Pdf (an analysis of the cytopathogenic effect of the virus after adding the Quercetin-Like Compounds). • Output analysis data statistic.pdf (Probit analysis: NSPE of the total with dose) • QTOF Water.jpg (Instrumentation for observing the LC-ESI MS) • Standard-Prof Laz carbon 1-2.jdf.pdf (Overlay standard carbon NMR) •
Figure 7-A-FS.jpg ( *In vitro* test of 0.05% quercetin-like compounds against live Newcastle disease virus at a dilution of 10 ^−5^ in chick kidney culture cells on 96-wells microplate. No cytopathogenic effect on culture cells was observed on magnified 40x by inverted microscope). •
Figure 7-B-FS.jpg ( *In vitro* test of 0.05% quercetin-like compounds against live Newcastle disease virus at a dilution of 10-4 in chick kidney culture cell on 96-wells microplate. Cytopathogenic effects were starting to appear on culture cells using an observed magnified 40x inverted microscope). •
Figure 7-original 600dpi (2).jpg (Observes well of microplate at test *in vitro*) • Standard-Prof LAZ-31 Jan 2023_carbon1-2jdf.pdf (Analysis carbon NMR standard). •
Figure 7-C-FS.jpg ( *In vitro* test of 0.05% quercetin-like compounds against live Newcastle disease virus at a dilution of 10-4 in chick kidney culture cell on 96-wells microplate. Cytopathogenic effects in cell culture were very clear in the spaces between cells that were still alive observed by an inverted microscope on magnified 40x). •
Figure 7-D-FS.jpg (Negative control of chicken kidney cell culture in Eagle’s medium grown on 96-wells microplate. Observed using 40x magnified of inverted microscope). • STANDAR-PROF LAZ-31_proton 1-3.jdf.pdf (An analysis proton NMR of Quercetin standard). •
Figure 7-E-FS.jpg (Positive control of chick kidney cell culture containing of live Newcastle disease virus at a dilution of 10 ^−1^) • Negative control.jpg (Control negative *in vitro* test) • STANDARD-PROF LAZ-31JAN2023_proton-1-3.jdf.pdf (Analysis proton NMR of quercetin). • STANDARD-PROF LAZ-31 JAN203_PROTON-1-3.JDF.PDF-PERB1.pdf (Analysis proton NMR of extract BD) • 13-June-1.jpg (Research activities) • 13-June-2.jpg (Research activities) • 13-June-3.jpg (Research activities) • 13-June-4.jpg (Research activities) • 21-June-1.jpg (Research activities) • 21-June-2.jpg (Research activities) • 21-June-13.jpg (Research activities) • 15-July-1.jpg (Research activities) • 15-July-2.jpg (Research activities) • 15-July-3.jpg (Research activities) • 15-July-5.jpg (Research activities) • 15-July-6.jpg (Research activities) • 20-July-13.jpg (Research activities) • 1-August-1.jpg (Research activities) • 1-August-2.jpg (Research activities) • 1-August-3.jpg (Research activities) • 1-August-4.jpg (Research activities) • 1-August-5.jpg (Research activities) • 4-august-10.jpg (Research activities) • 5-August-1.jpg (Research activities) • 5-August-2.jpg (Research activities) • 5-August-3.jpg (Research activities) • 5-August-4.jpg (Research activities) • 9-August-8.jpg (Research activities) • 9-August-9.jpg (Research activities) • 9-August-10.jpg (Research activities) • 9-August-11.jpg (Research activities) • 9-August-12.jpg (Research activities) • 9-August-25.jpg (Research activities) •
Figure 7 600dpi-edit New.jpg ( *In vitro* test with 500 μm scale) • 10-August-3.jpg (Research activities) • 10-August-4.jpg (Research activities) • 10-August-5.jpg (Research activities) • 10-August-6.jpg (Research activities) • 30-August-1.jpg (Research activities) • 31-August-1.jpg (Research activities) • Oct-1.jpg (Research activities) • Sept-1.jpg (Research activities) • Kontrol positif & AEI 600dpi (1).jpg. (Positive control) • Kontrol positif & AEI 600dpi (1).jpg (Copy positive control) •
*In vitro* analysis of quercetin-like compounds from mistletoe Dendrophthoe pentandra L. Miq as a potential antiviral agent for Newcastle disease.pdf (Webinar SATU) Data are available under the terms of the Creative Commons Attribution 4.0 International license (CC-BY 4.0).
