# Supplementary material for: In vitro analysis of quercetin-like compounds from mistletoe Dendrophthoe pentandra (L.) Miq as a potential antiviral agent for Newcastle disease
Source: F1000Res. 2024 Jul 23;12:1214. Originally published 2023 Sep 26. [Version 8] doi: 10.12688/f1000research.133489.8 (PMC11220444; doi:10.12688/f1000research.133489.8)
Supplement: Peer review history

# Reviewer report: Reviewer response for version 7

**DOI:** 10.5256/f1000research.168805.r300636

**Published:** 2024-07-10

**Authors:** Surat Hongsibsong

**Affiliations:** 1 Chiang Mai University, Thailand, Thailand

This version is approved

Is the work clearly and accurately presented and does it cite the current literature?

Yes

If applicable, is the statistical analysis and its interpretation appropriate?

I cannot comment. A qualified statistician is required.

Are all the source data underlying the results available to ensure full reproducibility?

No

Is the study design appropriate and is the work technically sound?

Partly

Are the conclusions drawn adequately supported by the results?

Partly

Are sufficient details of methods and analysis provided to allow replication by others?

Partly

Reviewer Expertise:

NA

I confirm that I have read this submission and believe that I have an appropriate level of expertise to confirm that it is of an acceptable scientific standard.

# Reviewer report: Reviewer response for version 6

**DOI:** 10.5256/f1000research.166738.r292944

**Published:** 2024-07-02

**Authors:** Surat Hongsibsong

**Affiliations:** 1 Chiang Mai University, Thailand, Thailand

The work shows an exciting objective. The methods should be written in detail. I have some suggestions for authors below.

 1. For The research on viruses, it would be nice if 1.1 authors provided the number of animal ethics approval certificates 1.2 The biosafety class, such as the laboratory class 3 or ???

 2. The method of intervention in vitro and cell experiments would be good if the author wrote some explanation about why the infected virus isolated from animals doesn't need to be compared with the standard virus group.

Is the work clearly and accurately presented and does it cite the current literature?

Yes

If applicable, is the statistical analysis and its interpretation appropriate?

I cannot comment. A qualified statistician is required.

Are all the source data underlying the results available to ensure full reproducibility?

No

Is the study design appropriate and is the work technically sound?

Partly

Are the conclusions drawn adequately supported by the results?

Partly

Are sufficient details of methods and analysis provided to allow replication by others?

Partly

Reviewer Expertise:

As mention in comment for authors

I confirm that I have read this submission and believe that I have an appropriate level of expertise to confirm that it is of an acceptable scientific standard, however I have significant reservations, as outlined above.

# Response

**Published:** 2024-07-03

**Authors:** Prof. Lazuardi Mochamad

**Affiliations:** Universitas Airlangga, Indonesia

Request 1.

 The work shows an exciting objective. The methods should be written in detail. I have some suggestions for authors below.

 For The research on viruses, it would be nice if 1.1 authors provided the number of animal ethics approval certificates 1.2 The biosafety class, such as the laboratory class 3 or ???

 Answer Response

 Page 13 first Alinea below:

  In the ethics exam, experimental animals were approved to use 30 chicken experimental animals for 6 subgroup divisions of 5 chickens per subgroup. The animal ethics clearance certificate was issued with No: 1.KEH.060.04.2023 on May 12 th, 2023. The implementation was under Biosafety Laboratory Level (BSL) III control. It was carried out according to operational procedures at the Infectious Tropical Diseases Research Center Universitas Airlangga (ITDRCUA) which has been approved as a type 3 BSL lab in Surabaya-Indonesia. Proof of laboratory predicate certificates can be obtained at the administration of ITDRCUA Mulyorejo Rd, Campus C, Universitas Airlangga, Surabaya-Indonesia. 60115.

 Request 2

 The method of intervention in vitro and cell experiments would be good if the author wrote some explanation about why the infected virus isolated from animals doesn't need to be compared with the standard virus group.

 Answer Response

 Page 4 first Alinea below:

 In this study, the virus isolated from infected chickens does not need to be compared with the standard ND virus because the character and level of virulence of the virus have been known from the research and laboratory records regarding the isolation at the Laboratory of Veterinary Pharmaceutical Sciences, Faculty of Veterinary Medicine, Universitas Airlangga. In addition, the virus is also known to have the character and degree of malignancy in other studies on ND viruses even though the target clinical subjects are different.

# Reviewer report: Reviewer response for version 5

**DOI:** 10.5256/f1000research.163209.r268001

**Published:** 2024-07-04

**Authors:** Asma Ashraf

**Affiliations:** 1 Government College University, Faisalabad, Pakistan

**Suggestion for Improvement**

 Clarify the experimental procedures and methodologies used in the in vitro investigations to enhance reproducibility and rigor.

 Provide more detailed discussions on the mechanisms underlying the antiviral activity of QLCs, including their interactions with NDV.

**Materials and Methods **

1. Geographical coordinates:

The format seems incorrect. Coordinates typically include degrees, minutes, and seconds. The given coordinates "3° 390 000 south, 103° 480 000 east" appear to be in a different format, possibly mixing degrees and decimal degrees. It's likely meant to be "3° 39' 00" S, 103° 48' 00" E" for the correct format. "3° 390 000 south" should be "3° 39' 00" S" for the correct format.

 "103° 480 000 east" should be "103° 48' 00" E" for the correct format. In the address, "Raya Jakarta – Bogor Km 46" seems odd. It might be more appropriate to mention a specific location or landmark instead of a kilometer marker. There's a typographical error in "misteltoe" which should be corrected to "mistletoe".

 In the sentence "Indonesian Consortium Research and Innovation National", "National" seems out of place. It might be intended to be "Institute" or "Center" instead of "National".

 The phrase "the collection of the samples started in March 2022 and continued until August 2022" is grammatically correct but could be improved for clarity. It might be clearer to say "Sampling began in March 2022 and lasted until August 2022".

**Qualitative flavonoid analysis**

 In the first method, "iron chloride(III)pasolution" is likely a typo. It should be "iron chloride(III) solution." The "Hydrochlorideacid" is a typo and should be corrected to "hydrochloric acid."

**Optimizing the mobile phase for column chromatography to separate the quercetin-like compounds**

 The term "polar solutions" is vague and could be more specific. It's unclear what is meant by "polar solutions" in this context. Are these polar solvents?

 The text mentions testing ratios of 4:6, 3:7, 2:8, and 1:9, but it's not clear what these ratios represent. Are they volume ratios of water to methanol?

Is the work clearly and accurately presented and does it cite the current literature?

Yes

If applicable, is the statistical analysis and its interpretation appropriate?

Yes

Are all the source data underlying the results available to ensure full reproducibility?

Yes

Is the study design appropriate and is the work technically sound?

Yes

Are the conclusions drawn adequately supported by the results?

Yes

Are sufficient details of methods and analysis provided to allow replication by others?

Yes

Reviewer Expertise:

Microbiology, Immunology Phytochemistry

I confirm that I have read this submission and believe that I have an appropriate level of expertise to confirm that it is of an acceptable scientific standard, however I have significant reservations, as outlined above.

# Response

**Published:** 2024-07-05

**Authors:** Prof. Lazuardi Mochamad

**Affiliations:** Universitas Airlangga, Indonesia

Request 1.

 Provide more detailed discussions on the mechanisms underlying the antiviral activity of QLCs, including their interactions with NDV.

 Add the sentences in Page 14 last paragraph as follows;

 The element QLCs in the form of 7-hydroxy-1-methoxy-2-methoxyxanthone are ligands with a phenolic part bound to a strong electronegative group that can resonate and interact with the substrate elements of viral base pairs that have a high-energy metabolism. At the time of the complexion, the strong electronegative binding of the ligand to the viral substrate was very strong. This property causes the ability of the virus to replicate very limited, resulting in the phenomenon of virucide.

 Request 2.

 Materials and Methods

 Geographical coordinates:

 Format already corrected  "3° 39' 00" S” and "103° 48' 00" E" for the correct format.

 Correction at below:

 The plant, as mistletoe, is *Dendrophthoe pentandra (L.) Miq.,* was identified by the Directorate Control of the Natural Science Collection, National Bureau of Innovative Research, official letter number B-1679/11.6.2/DI.05.07/6/2022, at the address of Raya Jakarta – Bogor, Cibinong 16911, on June 7 ^th^, 2022, with the Indonesian name BD. Sampling began in March 2022 and lasted until August 2022.

 Request 3

 Qualitative flavonoid analysis

 Correction in iron chloride(III) solution and hydrochloric acid.

 Request 4.

 Optimizing the mobile phase for column chromatography to separate the quercetin-like compounds

 The term "polar solutions" were described as follows:

 solution contains bonds between atoms that have very different electronegativities, which cause a dipole moment.  

 Corrected in sentences:  the fractions water: methanol as follows; 4:6, 3:7, 2:8, and 1:9 were tested.

# Response

**Published:** 2024-07-06

**Authors:** Prof. Lazuardi Mochamad

**Affiliations:** Universitas Airlangga, Indonesia

Reviewer III namely Asma determined a "typo" **Misteltoe** and must be change by **Mistletoe**, my manuscript did not words with the type **Misteltoe**

# Reviewer report: Reviewer response for version 5

**DOI:** 10.5256/f1000research.163209.r267998

**Published:** 2024-05-13

**Authors:** Jacinta Ngozi Omeke

**Affiliations:** 1 University of Nigeria, Nsukka, Nigeria

It is an intensive work but the materials and methods need some modifications. The normal standard method of re-isolation of the NDV after infection was not mentioned. There are reports in materials and Methods that supposed to be in Result section, e.g clinical signs of NDV presented by birds after infection.

 Moderate English editing is essential for e.g average endurance of infected chickens. The endurance period was the incubation period of the virus. (Recast the sentence).

 In Materials and methods line 16, bacterial should be separated from Fungi.

 In result, page 11, wavenumber should be separated. it was not uniformly written in many pages, (check page 12, 13).

 The low yield of the extract should be compared to early researchers. 

 In discussion, the authors should compare their results to other peoples works.

Is the work clearly and accurately presented and does it cite the current literature?

Partly

If applicable, is the statistical analysis and its interpretation appropriate?

I cannot comment. A qualified statistician is required.

Are all the source data underlying the results available to ensure full reproducibility?

Yes

Is the study design appropriate and is the work technically sound?

Partly

Are the conclusions drawn adequately supported by the results?

Partly

Are sufficient details of methods and analysis provided to allow replication by others?

Partly

Reviewer Expertise:

I specialized in Diagnostic  Pathology (Avian Pathologist)

I confirm that I have read this submission and believe that I have an appropriate level of expertise to confirm that it is of an acceptable scientific standard, however I have significant reservations, as outlined above.

# Response

**Published:** 2024-05-17

**Authors:** Prof. Lazuardi Mochamad

**Affiliations:** Universitas Airlangga, Indonesia

**Reviewer II**: The normal standard method of re-isolation of the NDV after infection was not mentioned.

**User comment: **It has been edited according to request in the virus (method) subchapter in the red sentence of the revised version of the Manuscript.

**Reviewer II:** There are reports in materials and Methods that supposed to be in Result section, e.g clinical signs of NDV presented by birds after infection.

**User comment**: It has been revised in the results section of the second paragraph, the sentence is colored red

**Reviewer II:** The endurance period was the incubation period of the virus. (Recast the sentence).

**User comment: **The sentences will be changed as follows: The average ability to survive infection with artificial infections of the NDV virus only lasted three days out of the five-day observation plan. (In red sentences)

**Reviewer II:** In Materials and methods line 16, bacterial should be separated from Fungi.

**User comment:** It has been revised (in red sentences)

**Reviewer II:** wavenumber should be separated. it was not uniformly written in many pages.

**User comment:** It has been revised (in red sentences)

**Reviewer II: **The low yield of the extract should be compared to early researchers.

**User Comment**: It has been revised (in red sentences) as follows: QLCs results are generally also obtained when the same isolation is carried out on secondary metabolites from other mistletoe leaves, with an average of 1 to 0.005%, especially mistletoe plant species. 25,33

**Reviewer II:** In discussion, the authors should compare their results to other peoples works

**User comment :** It has been revised (in red sentences) as follows: The research results that have been found, compared with other researchers even from different plant medicines, show that the ability of the secondary metabolites found is one and a half times to two times stronger as an anti-virus. ^2,5,17,19^

# Reviewer report: Reviewer response for version 5

**DOI:** 10.5256/f1000research.163209.r252061

**Published:** 2024-03-06

**Authors:** Adel M. Abdelaziz

**Affiliations:** 1 Zagazig University, Zagazig, Ash Sharqia Governorate, Egypt

There is no comment.

Is the work clearly and accurately presented and does it cite the current literature?

Yes

If applicable, is the statistical analysis and its interpretation appropriate?

Partly

Are all the source data underlying the results available to ensure full reproducibility?

Yes

Is the study design appropriate and is the work technically sound?

Partly

Are the conclusions drawn adequately supported by the results?

Partly

Are sufficient details of methods and analysis provided to allow replication by others?

Partly

Reviewer Expertise:

Avian disease and medicine, avian virology, molecular biology

I confirm that I have read this submission and believe that I have an appropriate level of expertise to confirm that it is of an acceptable scientific standard.

# Reviewer report: Reviewer response for version 4

**DOI:** 10.5256/f1000research.158930.r223638

**Published:** 2024-02-27

**Authors:** Adel M. Abdelaziz

**Affiliations:** 1 Zagazig University, Zagazig, Ash Sharqia Governorate, Egypt

The assessment of challenge strain virus is crucial point in this research work, because the potency of these plant extract as antiviral agent depend on the virulence and pathogenicity of this challenge viral strain. The evaluation of virulence of NDV strain used in challenge with QLCs from BD PLANTS must be applied with the standard methods.

**Method section:**

 The seed virus characterization and virulence evaluation must be applied using a standard method with references, or obtained from any previous research work. 

 -The method of egg inoculation not correct, change this sentence "  The NDV substances were propagated using chicken embryos in eggs (aged 4 to 5 days) by injecting 0.25 mL of the NDV substance onto **the eggshell until the needle was in the air cell part of the egg** ."  to be .......... the NDV was propagated using SPF embryonated chicken eggs (9-11 days old) **via allantoic cavity and icubated at 37 °C for 2–7 days according to OIE 2021.**

**Result section:**

 The researcher must use a standard strain for challenge. The modified field test model is not a standard method for evaluation of viral virulence, please added a standard method with reference.

Is the work clearly and accurately presented and does it cite the current literature?

Yes

If applicable, is the statistical analysis and its interpretation appropriate?

Partly

Are all the source data underlying the results available to ensure full reproducibility?

Yes

Is the study design appropriate and is the work technically sound?

Partly

Are the conclusions drawn adequately supported by the results?

Partly

Are sufficient details of methods and analysis provided to allow replication by others?

Partly

Reviewer Expertise:

Avian disease and medicine, avian virology, molecular biology

I confirm that I have read this submission and believe that I have an appropriate level of expertise to confirm that it is of an acceptable scientific standard, however I have significant reservations, as outlined above.

# Response

**Published:** 2024-02-27

**Authors:** Prof. Lazuardi Mochamad

**Affiliations:** Universitas Airlangga, Indonesia

Revised:

 Method Section

 In the 8 lines of the viruses subtopic (method section), a statement has been added that the virus is the same as the type of NDV virus that caused an outbreak in ducks one year ago, as follows;

 In 8 lines of the viruses’ subtopic (method section), a statement has been added that the virus is the same as the NDV type of virus that caused an outbreak in ducks one year ago. So the reference was changed, namely reference number 20.

 20. Naimah Putri, Rahayu Ernawati, Jola Rahmahani, et al: Phylogenetic relationship and genotype variation of six Newcastle disease viruses isolated from duck in Indonesia. Vet. World. 2019;14(1):276-284. 33642815 10.14202/vetworld.2021.276-284 PMC7896909.

 In the 34 lines of the viruses subtopic (method section), the statement has been revised at a request of the reviewer.

 Result Section

 The statement has been revised as requested by the reviewer by providing a statement that the identification and characterization of the NDV virus had been carried out by previous researchers using the same isolate when an outbreak in ducks occurred in the same place.

 The field virus isolate used in this study was identified as VO-expressed NDV with high levels of virulence (Table 2 and Table 3). Virus identification and characterization of the NDV virus followed previous research on cases of duck outbreaks in the same area and using the same isolates. ^20^^ ,^^22^

# Reviewer report: Reviewer response for version 3

**DOI:** 10.5256/f1000research.158444.r221458

**Published:** 2023-11-13

**Authors:** Adel M. Abdelaziz

**Affiliations:** 1 Zagazig University, Zagazig, Ash Sharqia Governorate, Egypt

The assessment of challenge strain virus is crucial point in this research work, because the potency of these plant extract as antiviral agent depend on the virulence and pathogenicity of this challenge viral strain. The evaluation of virulence of NDV strain used in challenge with QLCs from BD PLANTS must be applied with the standard methods.

**Method section:**

 The seed virus characterization and virulence evaluation must be applied using a standard method with references, or obtained from any previous research work. 

 The method of egg inoculation not correct, could you add any reference recommending this method of egg inoculation?

**Result section:**

 The researcher must use a standard strain for challenge. The modified field test model is not a standard method for evaluation of viral virulence, please added a standard method with reference.

Is the work clearly and accurately presented and does it cite the current literature?

Yes

If applicable, is the statistical analysis and its interpretation appropriate?

Partly

Are all the source data underlying the results available to ensure full reproducibility?

Yes

Is the study design appropriate and is the work technically sound?

Partly

Are the conclusions drawn adequately supported by the results?

Partly

Are sufficient details of methods and analysis provided to allow replication by others?

Partly

Reviewer Expertise:

Avian disease and medicine, avian virology, molecular biology

I confirm that I have read this submission and believe that I have an appropriate level of expertise to confirm that it is of an acceptable scientific standard, however I have significant reservations, as outlined above.

# Response

**Published:** 2023-11-14

**Authors:** Prof. Lazuardi Mochamad

**Affiliations:** Universitas Airlangga, Indonesia

Comment to Reviewer:

 Inoculate in eggs.

 The first propagation technique for live viruses using chicken egg embryos, since the author first worked in the Indonesia Veterinary Drugs Assay, Directorate General Livestock, and Animal Health in 1983 has always been practiced under the supervision of Japanese veterinary drugs assay staff and written in the book Indonesian Veterinary Drug Pharmacopoeia (Biologic) 2009.

 Standard virulence virus NDV.

 This study should not use other types of NDV virus standards other than the products of the Indonesia Veterinary Pharma Centre, because regulation from the Government does not allow to use of strains outside the products of the Indonesia Veterinary Pharma Centre for live viruses.

# Reviewer report: Reviewer response for version 2

**DOI:** 10.5256/f1000research.156703.r214625

**Published:** 2023-10-30

**Authors:** Adel M. Abdelaziz

**Affiliations:** 1 Zagazig University, Zagazig, Ash Sharqia Governorate, Egypt

The work is clear and sound as *in vitro* study of the antiviral agent, but the evaluation of antiviral activity of any compound need field trial *in vivo* by challenge test with field virus strain on vaccinated and non-vaccinated bird treated with the compound. The effect of these plant compound is to enhance the immunity either humoral or cell mediated immunity, in either vaccinated and non-vaccinated bird.

 By answering and addressing these observations, the research can be accepted. If they are not addressed, the research cannot be accepted:

**The field virus **

1. Challenge virus need to assist its velogenicity by mean death time (MDT) or other known pathogenicity index as (IVPI OR ICPI).
2. Do you have any data or another method about assessment of virulene of field virus strain?
3. Do you have any reference about the pathogenicity assessment of field virus?
4. Do you have any reference about (Modified field test)? If present please put it.
5. Do you have any reference for the method of egg injection method? Or correct it.

**In abstract**

1. Remove the word 'and in, "...and in chicken kidney cell culture."
2.  Correct the word 'virulency' in abstract to be, 'virulence'.
3. Correct the conclusion section in abstract as follows: "...QLCs from flavonoids from the leaves of BD have invetro antiviral bioactivity against NDVs against the NDV at a virulence titer of 10 ^−5^ Tissue Culture Infectious Dose 50% (TCID50) in chicken kidney cell culture. QLCs may have the potential to be developed as medicinal compounds for the treatment of other human or animal viral infections."

Is the work clearly and accurately presented and does it cite the current literature?

Yes

If applicable, is the statistical analysis and its interpretation appropriate?

Partly

Are all the source data underlying the results available to ensure full reproducibility?

Yes

Is the study design appropriate and is the work technically sound?

Partly

Are the conclusions drawn adequately supported by the results?

Partly

Are sufficient details of methods and analysis provided to allow replication by others?

Partly

Reviewer Expertise:

Avian disease and medicine, avian virology, molecular biology

I confirm that I have read this submission and believe that I have an appropriate level of expertise to confirm that it is of an acceptable scientific standard, however I have significant reservations, as outlined above.

# Response

**Published:** 2023-11-02

**Authors:** Prof. Lazuardi Mochamad

**Affiliations:** Universitas Airlangga, Indonesia

Comment:

 1. Challenge virus need to assist its velogenicity by mean death time (MDT) or other known pathogenicity index as (IVPI OR ICPI).

 Response: 

 The data of the virulence test of the field virus had been described in Table 2 and Table 3

 2.Do you have any data or another method about assessment of virulene of field virus strain?

 Response:

 This research did not screen the strain yet, but for vaccination and to challenge the live virus we used a homologous strain as an Indonesia PUSVETMA Strain of NDV at titer HA and HI assessed method. The new references at 36 was added to the list of references. 

 3. Do you have any reference for the pathogenicity assessment of field virus?

 Response:

 The new reference at 37 and 38

 4. Do you have any reference about (Modified field test)? If present please put it.

 Response:

 This has been described in Suggest of this research parts. 

 5.  Do you have any reference for the method of egg injection method? Or correct it.

 Response:

 Will be described in the Suggest of this research part and add the new reference at no. 49 and 50
